# Ogerin mediated inhibition of TGF-β(1) induced myofibroblast differentiation is potentiated by acidic pH

**DOI:** 10.1371/journal.pone.0271608

**Published:** 2022-07-28

**Authors:** Tyler J. Bell, David J. Nagel, Collynn F. Woeller, R. Mathew Kottmann

**Affiliations:** 1 Department of Environmental Medicine Toxicology Training Program, University of Rochester School of Medicine and Dentistry, Rochester, NY, United States of America; 2 Department of Pulmonary and Critical Care Medicine, University of Rochester School of Medicine and Dentistry, Rochester, NY, United States of America; 3 Department of Ophthalmology, University of Rochester School of Medicine and Dentistry, Rochester, NY, United States of America; Medical University of South Carolina, UNITED STATES

## Abstract

Transforming growth factor beta (TGF-β) induced myofibroblast differentiation is central to the pathological scarring observed in Idiopathic Pulmonary Fibrosis (IPF) and other fibrotic diseases. Our lab has recently identified expression of GPR68 (Ovarian Cancer Gene Receptor 1, OGR1), a pH sensing G-protein coupled receptor, as a negative regulator of TGF-β induced profibrotic effects in primary human lung fibroblasts (PHLFs). We therefore hypothesized that small molecule activators of GPR68 would inhibit myofibroblast differentiation. Ogerin is a positive allosteric modulator (PAM) of GPR68, inducing a leftward shift of the dose response curve to proton induced signaling. Using PHLFs derived from patients with both non-fibrotic and IPF diagnoses, we show that Ogerin inhibits, and partially reverses TGF-β induced myofibroblast differentiation in a dose dependent manner. This occurs at the transcriptional level without inhibition of canonical TGF-β induced SMAD signaling. Ogerin induces PKA dependent CREB phosphorylation, a marker of Gα_s_ pathway activation. The ability of Ogerin to inhibit both basal and TGF-β induced collagen gene transcription, and induction of Gα_s_ signaling is enhanced at an acidic pH (pH 6.8). Similar findings were also found using fibroblasts derived from dermal, intestinal, and orbital tissue. The biological role of GPR68 in different tissues, cell types, and disease states is an evolving and emerging field. This work adds to the understanding of Gα_s_ coupled GPCRs in fibrotic lung disease, the ability to harness the pH sensing properties of GPR68, and conserved mechanisms of fibrosis across different organ systems.

## Background

Fibrotic diseases represent a major unmet healthcare need worldwide. Up to 45% of deaths in the developed world are due in part to diseases with fibrotic characteristics [1]. While there are distinct initiating events, pathophysiology, progression, prognosis, inflammatory status, and treatment approaches for each disease and organ system, there are common underlying biological mechanisms. Central to fibrotic diseases is transforming growth factor beta-1 (TGF-β) induced myofibroblast differentiation [1–11].

Myofibroblasts were first described in the dermis [12]. Knowledge of their role in normal wound healing responses, scarring, and fibrotic pathologies throughout the body has grown immensely, but remains incomplete. Scarring of the dermis, such as hypertrophic and keloid scars or those arising in scleroderma, have limited treatment options and can cause significant psychological and physiological issues [5,10]. Central to the pathology of Graves’ ophthalmology (Thyroid eye disease, TED), specifically type II disease, is excessive fibroblast activation akin to myofibroblast differentiation resulting in the excessive deposition of extracellular matrix (ECM) [13–17]. End-stage Crohn’s disease and ulcerative colitis can all progress to fibrosis with a known role of TGF-β and subepithelial myofibroblast like cells [18–21]. As such, inhibition of TGF-β induced myofibroblast differentiation is a central target for the treatment of fibrotic pathologies throughout the body.

Idiopathic Pulmonary Fibrosis (IPF) is a devastating disease in which deposition of scar tissue progressively obliterates the fine alveolar architecture of the lung and leads to respiratory failure [22,23]. While two small molecule pharmacological therapies currently have FDA approval for the treatment of IPF (Nintedanib and Pirfenidone), no therapies reverse the progressive accumulation of scar tissue or restore loss of lung function [24,25]. Nintedanib and Pirfenidone slow the rate of lung function decline in patients with IPF [24,26,27] and their efficacy due at least in part through the inhibition of myofibroblast differentiation [28–37]. Thus, there is an urgent need to expand our understanding of the role of myofibroblast differentiation in the pathogenesis of IPF and other fibrotic diseases and develop new therapeutic targets.

Although multiple cell types of the lung participate in the development and progression of IPF, current paradigms recognize the fibroblast as a key mediator of disease progression [23,26,38–40]. One histopathological hallmark of IPF is the emergence of alpha-Smooth Muscle Actin (αSMA) expressing myofibroblasts [41]. Myofibroblasts primarily arise through differentiation of resident fibroblasts [42–44] although other sources such as alveolar epithelial progenitors and fibrocytes are recognized [45–47]. Myofibroblast differentiation is primarily driven through incessant production and activation of the cytokine TGF-β [40,48,49]. TGF-β is elevated in the lungs of patients with IPF [50] and is sufficient to induce pulmonary fibrosis [51]. Differentiated myofibroblasts secrete increased levels of ECM proteins, the most abundant of which is collagen, contributing to the deposition of scar tissue in IPF and other fibrotic diseases [52].

TGF-β induced myofibroblast differentiation promotes metabolic reprogramming. Through increased expression of lactate dehydrogenase 5 (LDH5), increased glycolytic flux, and subsequent export of lactate, the extracellular space becomes acidified [53]. Subsequent to this finding, we became interested in how fibroblasts sense and respond to changes in extracellular pH. We identified expression of GPR68 as a significant negative regulator of myofibroblast phenotypes [54]. We subsequently hypothesized that activation of GPR68 would also inhibit TGF-β induced myofibroblast differentiation.

GPR68, also known as Ovarian Cancer Gene Receptor 1 or OGR1, is a proton sensing G-Protein Coupled Receptor (GPCR) [55–58]. The biological functions of GPR68 and other “orphan GPCRs” are largely unknown. Until recently, mechanistic interrogation of proton sensing GPCRs has been hindered by a lack of specific agonistic and antagonistic ligands [59]. GPR68 is silent at physiological pH (7.4). In response to acidification of the extracellular space, an increase in extracellular protons, GPR68 becomes active with a maximal response elicited at a pH of 6.8 [59]. Proton induced GPR68 activation is capable of coupling to multiple canonical G-protein signaling pathways in different cell types and contexts [58,60–67]. Recently, the small molecule Ogerin was identified as a positive allosteric modulator (PAM) of GPR68 [68–71]. In the presence of Ogerin, GPR68 becomes active at physiological pH and exhibits enhanced receptor activity as extracellular pH decreases [68]. Ogerin potentiates proton induced GPR68 activation of the Gα_s_ pathway, and inhibits proton induced GPR68 activation of the Gα_q_ pathway [68].

Substantial evidence supports activation of the Gα_s_ pathway inhibits TGF-β induced pro-fibrotic fibroblast phenotypes [72]. Activation of Gα_s_ coupled GPCRs elevates intracellular concentrations of cyclic adenosine 3’,5’-monophosphate (cAMP) through induction of adenylyl cyclase (AC) activity. Increased intracellular cAMP concentrations inhibit a multitude of pro-fibrotic fibroblast phenotypes including proliferation, myofibroblast differentiation, and production of scar contributing extracellular matrix components including collagen [73]. Activation of the Gα_s_ pathway by prostaglandin E_2_ (PGE_2_), prostacyclin Receptor (IP) agonists, dopamine receptor D1 agonists, and the direct stimulation of AC by forskolin, all inhibit myofibroblast differentiation [28,74–79]. While activation of the Gα_s_ pathway exerts antifibrotic effects in fibroblasts, the mechanism through which it antagonizes TGF-β induced myofibroblast differentiation is less clear.

We hypothesized that Ogerin would inhibit TGF-β induced myofibroblast differentiation. Further, we hypothesized that due to the pH sensing properties of GPR68, both the inhibition of myofibroblast differentiation and the activation of the Gα_s_ pathway by Ogerin would be enhanced in acidic pH. Our work adds to the growing body of literature that highlights Gα_s_ coupled GPCRs as potential therapeutic targets for IPF, and other fibrotic diseases. GPR68 may represent a critical regulator of fibroblast function and serve as a therapeutic target for fibrotic diseases throughout the body.

## Materials and methods

Detailed experimental designs, protocols, reagents, and materials are provided in S2 Methods. In brief, PHLFs were isolated from lung tissue as previously described [53,80,81], and other cell lines were provided by collaborators and are previously described. All tissue was obtained under informed, written consent prior to collection of lung tissue and under the approval of the Institutional Review Board of the University of Rochester RSRB protocol 00000014. An example of the consent form is provided in S12 Consent. The diagnosis of IPF was confirmed using the standard American Thoracic Society pathologic criteria [82]. Recombinant Human TGF-beta 1 protein (R&D Systems), Ogerin (Sigma-Aldrich), or the vehicle (DMSO) were added to media (MEM + 0.5% FBS) at concentrations, duration, and analyzed as indicated in figure legends. Statistical analysis was performed for each dataset using GraphPad Prism. An ordinary one-way ANOVA with Tukey’s post hoc test for multiple comparisons were used with *p* < 0.05 considered significant indicated with (*). ^ = *p* < 0.05, by Two -Way ANOVA with Multiple Comparisons, # = *p* < 0.05 by unpaired T-test, and *NS* = Not Significant. All data expressed as indicated on plots and figure legends with individual technical replicates with range or means +/- SEM.

## Results and discussion

### Results

#### Ogerin inhibits and partially reverses TGF-β induced pro-fibrotic fibroblast phenotypes

**Fig 1:**
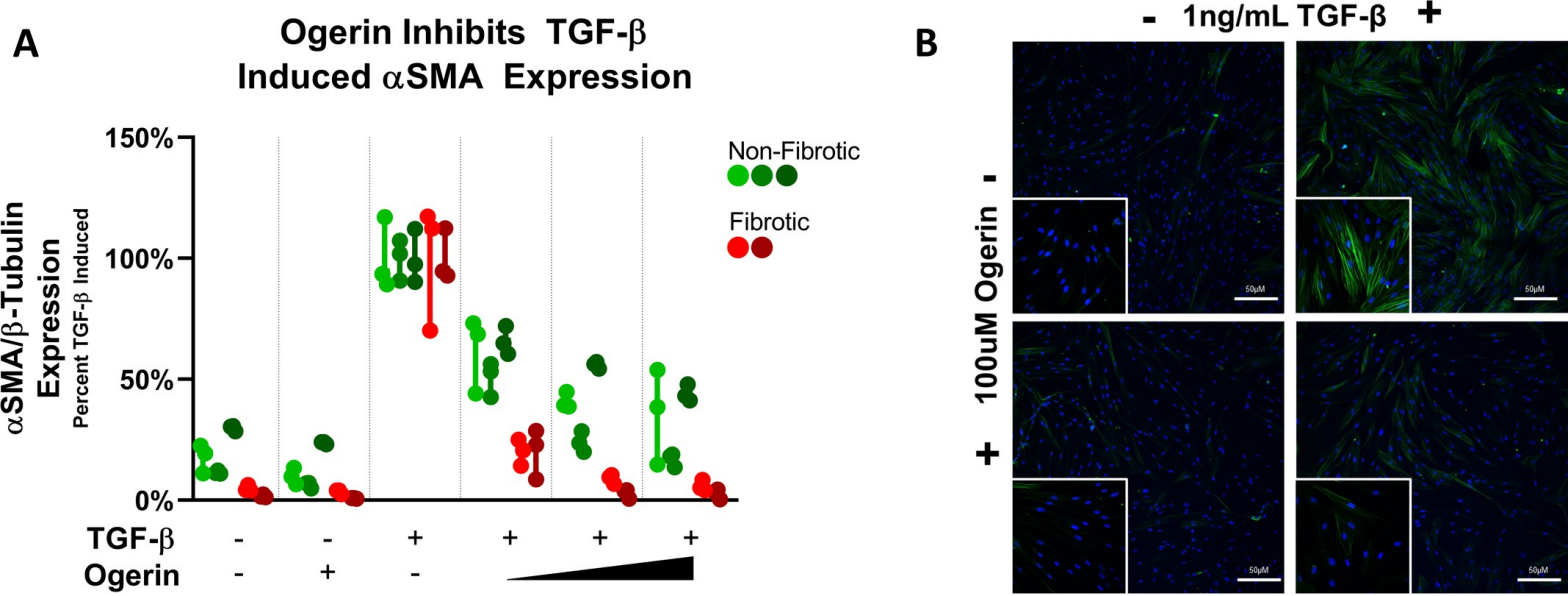
Ogerin inhibits TGF-β induced myofibroblast differentiation in primary human lung fibroblasts in a dose dependent manner. A: Primary human lung fibroblasts derived from donors with non-fibrotic or IPF diagnosis co-treated with 1ng/mL TGF-β and/or 50–150μM Ogerin for 72 hours. Whole cell lysates were analyzed for αSMA expression level via Western Blot. αSMA Band expression standardized to β-Tubulin, expressed as percent TGF-β induced. n = 3 per donor per treatment group, dots representative of technical replicates with bars representative of technical replicate range. Statistics omitted for clarity, individual plots with statistical analysis provided in S1 Fig. B: Primary human lung fibroblasts (Non-Fibrotic Donor #1) grown on glass microscope slides co-treated with 1ng/mL TGF-β and/or 100μM Ogerin for 72 hours. Cells fixed, permeabilized and stained for αSMA (Green) and nuclei (DAPI, Blue). Representative confocal images with 50μM scale bar shown in 200x image, and 400x inset. Full dataset provided in S2 Fig.

To ensure our *in vitro* models were relevant across disease state and naturally occurring biological variability, we utilized PHLFs from fibrotic and non-fibrotic donors. PHLFs were treated with 1ng/mL of TGF-β to induce myofibroblast differentiation and co-treated with or without 50–150μM Ogerin for 72 hours. Ogerin inhibited TGF-β induced αSMA expression in a dose dependent manner in all cell lines as shown by Western blot (Fig 1A) and immunofluorescent (IF) microscopy (Fig 1B). Although TGF-β induced a marked increase αSMA in all cell lines, the degree to which αSMA expression was upregulated from baseline was more marked in PHFLs from donors with IPF (S1 Fig). Despite a more robust response to TGF-β stimulation, fibrotic cell lines responded more significantly to lower doses of Ogerin compared to PHLFs derived from non-fibrotic donors.

**Fig 2:**
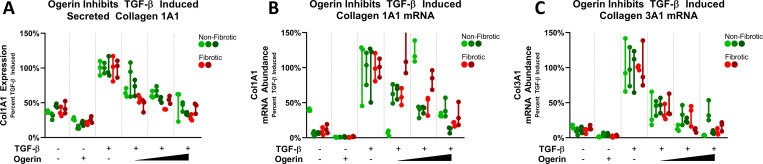
Ogerin inhibits TGF-β induced collagen production in primary human lung fibroblasts at the transcriptional level. A: Primary human lung fibroblasts derived from non-fibrotic or IPF patients were co-treated with 1ng/mL TGF-β and/or 50–150μM Ogerin for 72 hours. Cell culture supernatant was analyzed for secreted Col1A1 via slot blot. Band densitometry was standardized as percent TGF-β induced. n = 3 per donor per treatment group, bars representative of technical replicate range. Statistics omitted for clarity, individual plots with statistical analysis provided in S3B and S3C Fig. Primary human lung fibroblasts derived from non-fibrotic or IPF patients co-treated with 1ng/mL TGF-β and/or 50–150μM Ogerin for 48 hours. Col1A1 and Col3A1 mRNA transcript levels analyzed via qRT-PCR. Transcript abundance standardized to 18s rRNA by the ΔΔCt method and expressed as percent TGF-β induced. Statistics omitted for clarity, individual plots with statistical analysis provided in S4 and S5 Figs.

With the accumulation of myofibroblasts, IPF is characterized by the accumulation of excess matrix components, particularly collagen [41]. Recent advances in single cell RNA sequencing have further solidified fibroblasts as the primary cell type responsible for the overproduction of collagen in IPF [83–85]. To measure TGF-β induced collagen production, cell culture supernatant was analyzed for relative Collagen 1 abundance by slot blot. In all PHLFs cell lines following 72 hours of treatment with 1ng/mL of TGF-β, collagen secretion was significantly increased and inhibited in a dose dependent manner by co-treatment with 50–150μM Ogerin. Further, Ogerin also downregulated basal collagen secretion levels (Fig 2A).

Collagen production and secretion is regulated at the level of transcription, translation, and/or post translational modifications [86–88]. To identify how Ogerin inhibits collagen production, Col1A1 and Col3A1 mRNA transcript abundance was assessed by quantitative reverse transcription polymerase chain reaction (qRT-PCR). TGF-β induced Col1A1 and Col3A1 mRNA levels in all PHLF cell lines and was inhibited by Ogerin in a dose dependent manner (Fig 2B and 2C). In the absence of TGF-β, Ogerin also down-regulated baseline collagen transcript abundance. Collectively, these data demonstrate that Ogerin inhibits basal and TGF-β induced collagen production at the transcriptional level.

**Fig 3:**
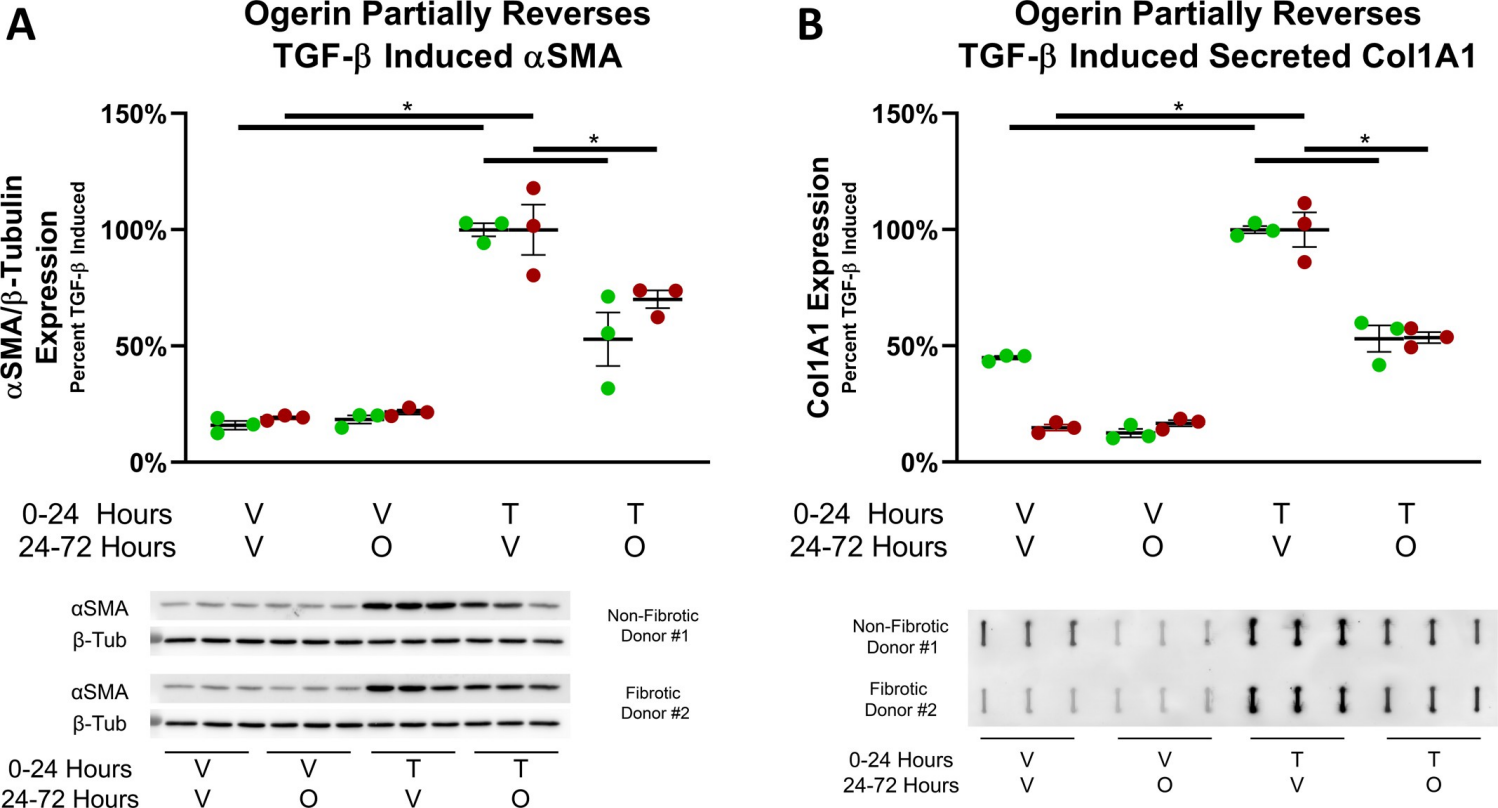
Ogerin partially reverses an established, TGF-β induced myofibroblast phenotype in primary human lung fibroblasts. Primary human lung fibroblasts derived from non-fibrotic or IPF patients were treated +/- 1ng/mL TGF-β (T) or Vehicle (V) for 24 hours. After 24-hour incubation, TGF-β containing media was removed and cells were rinsed with PBS. From 24–72 hours, cells were treated +/- 150μM Ogerin (O) or DMSO Vehicle (V). A: Whole cell lysates were analyzed for αSMA expression level via Western Blot. αSMA band expression was standardized to β-Tubulin, expressed as a percent of TGF-β induced. n = 3 per donor per treatment group, dots representative of technical replicates with SEM. * = p < 0.05 by One Way ANOVA with Tukey’s Post-Hoc Test for Multiple Comparisons. B: Cell culture supernatant was analyzed for secreted Col1A1 via slot blot. Band expression was standardized as a percent of TGF-β induced. n = 3 per donor per treatment group, dots representative of technical replicates with SEM. * = p < 0.05 by One-Way ANOVA with Tukey’s Post-Hoc Test for Multiple Comparisons.

While myofibroblast differentiation is often considered a terminal event in the development of fibrosis [89,90], this notion has been recently challenged [75,91,92]. To investigate whether Ogerin reverses an established myofibroblast phenotype, PHLFs were stimulated with TGF-β for 24 hours to induce myofibroblast differentiation. After this treatment period, TGF-β containing media was removed and replaced with media with Ogerin or the vehicle control for an additional 48 hours. Thus, externally applied TGF-β was only present for the first 24 hours of the experimental paradigm. Treatment with TGF-β for 24 hours was sufficient to induce a robust myofibroblast phenotype hallmarked by increased expression of αSMA and secretion of collagen into the cell culture supernatants. The application of Ogerin for the 24–72 hour treatment period significantly inhibited αSMA expression and collagen secretion (Fig 3A and 3B). These data demonstrate that Ogerin partially reverses myofibroblast phenotypes in PHLFs.

**Fig 4:**
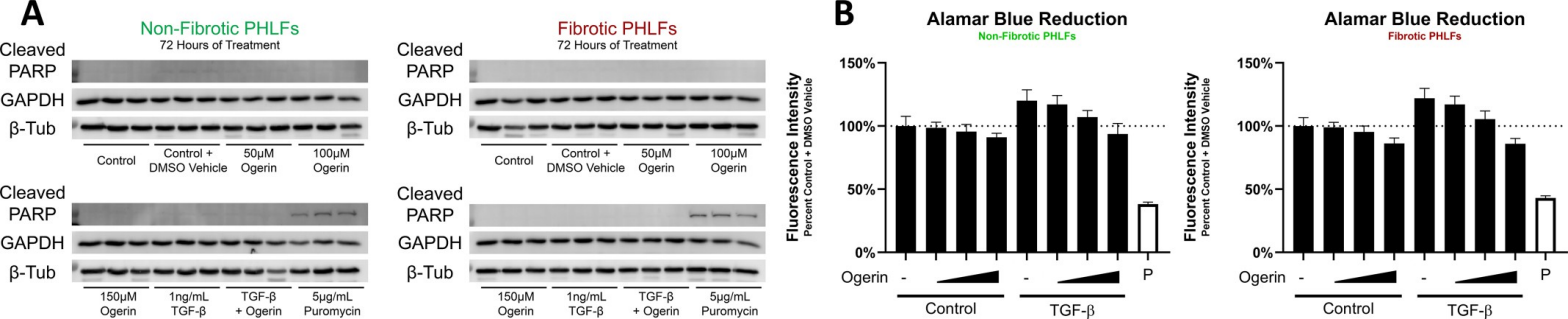
Prolonged treatment of Ogerin in the presence or absence of TGF-β does not induce apoptosis or negatively impact cellular viability. A: Primary human lung fibroblasts derived from non-fibrotic or IPF patients were treated +/- DMSO Vehicle, +/- 1ng/mL TGF-β, +/- 50–150μM Ogerin, or 5 μg/mL Puromycin (P) for 72 hours. Whole cell lysates were analyzed for cleaved PARP expression level via Western Blot with GAPDH and β-Tubulin as loading controls, n = 3 per donor per treatment group. B: Primary human lung fibroblasts derived from non-fibrotic or IPF patients were treated with the following reagents: +/- DMSO Vehicle, +/- 1ng/mL TGF-β, +/- 50–150μM Ogerin, or 5 μg/mL Puromycin for 72 hours. Following this incubation period, Alamar Blue cell viability reagent was added and allowed to incubate for 4 hours. Fluorescence intensity (Excitation 535nm, Emission 595nm) measured and expressed as a percent of DMSO Vehicle control. n = 8 per donor per treatment group, bars representative of technical replicate average +/- SEM. Representative images of cells following 72-hour treatment available in S6 Fig.

To ensure the previously described results were not the result of a toxic effect of Ogerin, multiple approaches were utilized. Visual inspection of fibroblast cell cultures using brightfield polarized light microscopy following 72 hours of treatment with Ogerin indicated no overt changes in morphology, adhesion, or density relative to the vehicle control. Treatment with TGF-β induced hallmark morphological changes associated with myofibroblast differentiation which were inhibited with co-treatment with Ogerin (S6 Fig). To determine whether Ogerin induced fibroblast apoptosis, the presence of cleaved poly(ADP-ribose) polymerase PARP [93,94] was measured by Western blot following a 72-hour treatment period with puromycin used as a positive control. Ogerin did not induce cleaved PARP in PHLFs (Fig 4A). To quantitatively assess cellular viability, an Alamar Blue assay, an indicator of cellular viability and proliferation based on the reducing capacity of cells, was performed after a 72-hour treatment period. There were no significant decreases in viability of the Ogerin treated cells relative to the vehicle control. Treatment with TGF-β increased reduction of Alamar Blue, which was inhibited with increasing concentrations of Ogerin (Fig 4B). These results suggest that Ogerin inhibits TGF-β stimulated proliferation [95,96].

#### Ogerin inhibits TGF-β induced gene transcription in a SMAD reporter construct but does not inhibit SMAD3 phosphorylation, nuclear translocation, or chromatin binding

**Fig 5:**
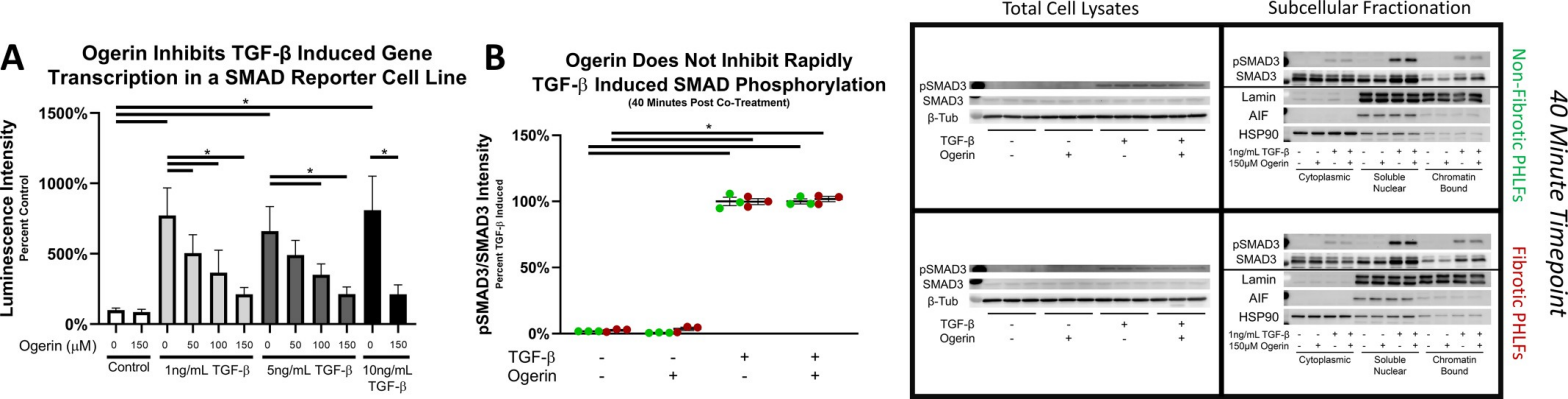
Ogerin inhibits TGF-β induced gene transcription in a SMAD reporter assay. Ogerin does not inhibit TGF-β induced SMAD3 phosphorylation, nuclear localization, or chromatin binding in primary human lung fibroblasts. A: HEK293 cells stably transfected with a TGF-β responsive luciferase element under the transcriptional control of upstream SMAD binding elements co-treated for 24 hours +/- 1-10ng/mL TGF-β and/or +/- 50–150μM Ogerin. Cells were lysed with luciferase substrates and measured for luminescence intensity, expressed as a percent of control. n = 8 per treatment group, bars representative of technical replicate average +/- SEM. B: Primary human lung fibroblasts derived from non-fibrotic and IPF patients were co-treated with 1ng/mL TGF-β and/or 150μM Ogerin for 40 minutes. Whole cell lysates were analyzed for phosphorylated SMAD3 expression via Western Blot. Phosphorylated SMAD3 band intensity was standardized to total SMAD3 band intensity and expressed as a percent of TGF-β induced. n = 3 per donor per treatment group, dots representative of technical replicates with SEM. Cytoplasmic, soluble nuclear, and chromatin bound subcellular fractionations analyzed for total and phosphorylated SMAD3, and for fractionation efficiency utilizing the same samples on a parallel blot. n = 1 per treatment group per fraction. * = p < 0.05 by One-Way ANOVA with Tukey’s Post-Hoc Test for Multiple Comparisons.

To elucidate the potential mechanism(s) through which Ogerin inhibits myofibroblast differentiation and TGF-β induced gene transcription, canonical TGF-β induced SMAD signaling was interrogated. A TGF-β responsive reporter construct (SB10) was utilized to characterize the effects of Ogerin on SMAD regulated gene transcription. As previously described [97], the reporter construct contains four tandem SMAD binding Elements (SBEs) upstream of the minimal thymidine kinase promoter which controls transcription of the firefly luciferase (Luc2P). Treatment of the reporter line with TGF-β for 24 hours robustly increases transcription of the luciferase as measured by luminescence intensity. Similar to the effects observed in PHLFs, Ogerin inhibited TGF-β induced luciferase activity in a dose dependent manner (Fig 5A) further indicating that Ogerin inhibits myofibroblast differentiation through inhibition of TGF-β induced gene transcription.

To determine whether Ogerin inhibits SMAD signaling dynamics [98–101], phosphorylation, translocation, and chromatin binding, was assessed. As expected, TGF-β induced SMAD3 phosphorylation in PHLFs from both Non-Fibrotic and Fibrotic donors in whole cell lysates. Ogerin, applied as a co-treatment (Fig 5B) or as a 1-hour pre-treatment (S7 Fig) did not inhibit TGF-β induced SMAD3 phosphorylation in whole cell lysates. To determine if other aspects of SMAD signaling were inhibited in the presence of Ogerin, cytoplasmic, nuclear, and chromatin bound subcellular fractions were isolated from PHLFs treated with TGF-β and/or Ogerin. Fractionation efficiency was confirmed with heat shock protein 90 (HSP90) primarily observed in the cytoplasmic fraction [102], and Lamin proteins restricted to the soluble and chromatin bound nuclear fractions [103]. TGF-β induced nuclear localization and chromatin binding of total and phosphorylated SMAD3 protein which was not significantly inhibited by Ogerin (Fig 5B). Collectively these results indicate that while Ogerin inhibits TGF-β induced gene transcription, receptor activation, SMAD3 phosphorylation, nuclear localization, and chromatin binding of SMAD3 are unaffected.

#### Ogerin activates Gα_s_ signaling in PHLFs

**Fig 6:**
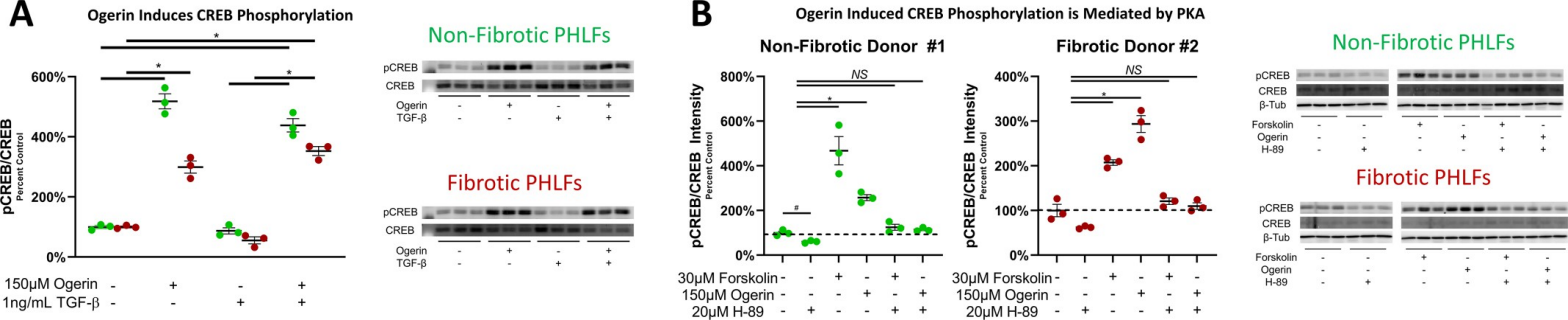
Ogerin induces Gα_s_ signaling as evident by PKA dependent CREB phosphorylation. A: Primary human lung fibroblasts derived from non-fibrotic or IPF patients were co-treated with 1ng/mL TGF-β and/or 150μM Ogerin for 40 Minutes. Whole cell lysates analyzed for phosphorylated CREB expression via Western Blot. Phosphorylated CREB intensity was standardized to total CREB expressed as a percent of control. n = 3 per donor per treatment group, dots representative of technical replicates with SEM. B: Primary human lung fibroblasts derived from non-fibrotic or IPF patients pre-treated with 20μM of the PKA Inhibitor H-89 for 30 minutes followed by +/- 30μM Forskolin or +/- 150μM Ogerin for 40 Minutes. CREB phosphorylation was analyzed as described for 6A. * = p < 0.05, NS = Not Significant by One -Way ANOVA with Tukey’s Post-Hoc Test for Multiple Comparisons.

We next sought to determine the effect of Ogerin on canonical GPCR intracellular signaling in PHLFs. In HEK293 cells overexpressing GPR68, Ogerin induces Gα_s_ intracellular signaling measured by increases in intracellular cAMP [68]. Ogerin acts as a biased agonist [64,104,105] preferentially stimulating Gα_s_ over Gα_q_ (measured by intracellular Ca^2+^ levels) [68]. Levels of phosphorylated CREB (pCREB), a downstream mediator of Gα_s_ signaling [28,106], were measured by Western blot and standardized to total CREB levels. Ogerin induced CREB phosphorylation in both Non-Fibrotic and Fibrotic PHLFs. The induction of CREB phosphorylation was not significantly altered by the presence of TGF-β (Fig 6A).

While CREB phosphorylation at serine-133 is a well-established marker of PKA activation, we sought to ensure our observations of Ogerin induced CREB phosphorylation were dependent upon PKA. PHLFs were pre-treated with the PKA inhibitor H-89 for 30 minutes, followed by a 40-minute treatment of Ogerin or Forskolin (a direct activator of AC). As previously observed and expected, Ogerin and Forskolin induced CREB phosphorylation. However, when PHLFs were pretreated with H-89, neither Ogerin nor Forskolin induced CREB phosphorylation (Fig 6B). Thus, Ogerin induces CREB phosphorylation through activation of PKA and suggests that Ogerin activates canonical Gα_s_ signaling in PHLFs as has been observed in other cell types [68–71].

#### Ogerin’s activation of Gα_s_ signaling and inhibition of collagen gene transcription is potentiated by acidic pH

**Fig 7:**
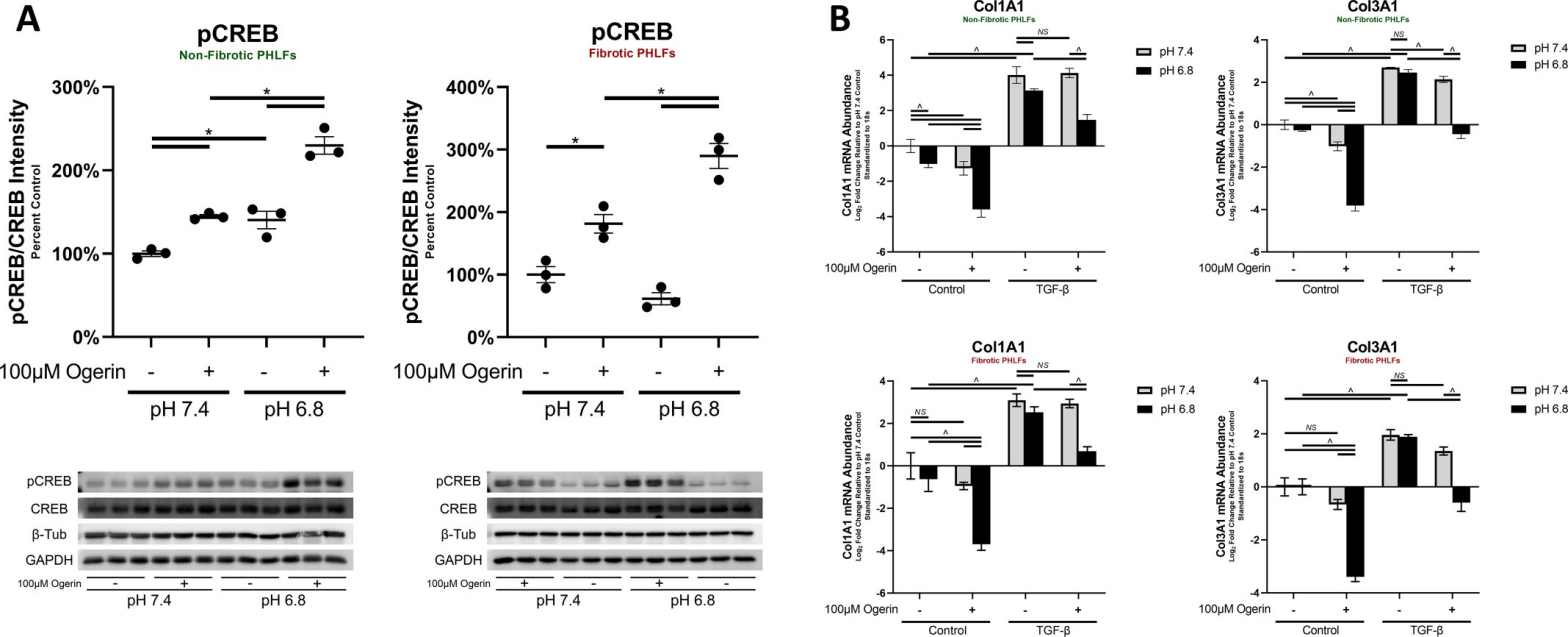
Ogerin’s activation Gα_s_ signaling and inhibition of basal and TGF-β induced collagen gene transcription is potentiated by acidic pH. A: Primary human lung fibroblasts derived from non-fibrotic or IPF patients were treated +/- 100μM Ogerin for 40 Minutes in pH adjusted PBS. Whole cell lysates were analyzed for phosphorylated CREB expression via Western Blot. Phosphorylated CREB intensity standardized to total CREB expressed as a percent of control. n = 3 per donor per treatment group, dots representative of technical replicates with SEM. B: Primary human lung fibroblasts derived from non-fibrotic subjects or those with IPF were co-treated with 1ng/mL TGF-β and/or 100μM Ogerin for 48 hours in pH adjusted, CO_2_ Independent media. Col1A1 and Col3A1 mRNA transcript levels were analyzed via qRT-PCR. Transcript abundance was standardized to 18s rRNA by the ΔΔCt method and expressed as percent TGF-β induced. ^ = p < 0.05, NS = Not Significant by Two -Way ANOVA with Multiple Comparisons of cells means regardless of rows and columns. * = p < 0.05, by One-Way ANOVA with Tukey’s Post-Hoc Test for Multiple Comparisons.

In other cell types, activation of Gα_s_ signaling by Ogerin is potentiated by acidic extracellular pH [68,69,71]. To investigate the effect of physiologically and pathologically relevant extracellular pH levels [107] on Ogerin induced Gα_s_ signaling, CREB phosphorylation was assessed at a pH of 7.4 and 6.8 in the presence and absence of 100μM Ogerin. To ensure the any observed effect of Ogerin on CREB phosphorylation was due solely to changes in pH and not due to the effect of acidic pH on media components, these experiments were performed in pH adjusted PBS. The induction of CREB phosphorylation by Ogerin was significantly potentiated at a pH of 6.8 relative to a pH of 7.4 in both Non-Fibrotic and Fibrotic PHLFs. In Non-Fibrotic PHLFs, acidic pH in the absence of Ogerin also induced CREB phosphorylation (Fig 7A). In pH adjusted CO_2_ independent media, the enhanced effect of Ogerin on Gα_s_ signaling was also evident (S8 Fig).

Culture systems utilizing CO_2_ independent media were further utilized to investigate the effect of Ogerin in acidic extracellular pH conditions on TGF-β induced gene transcription. In the absence of TGF-β, Ogerin induced downregulation of basal Collagen gene transcription as was previously observed, and was significantly potentiated in acidic (6.8) compared to physiological (7.4) pH. The level to which TGF-β induced collagen mRNA was equivalent at both physiological and acidic pH, however the degree to which this was inhibited by Ogerin was significantly potentiated at an acidic pH (Fig 7B). Together, these results indicate that both Ogerin induced Gα_s_ signaling and inhibition of collagen gene transcription are potentiated by acidic pH.

#### Ogerin inhibits myofibroblast differentiation in human dermal, intestinal, and orbital fibroblasts

**Fig 8:**
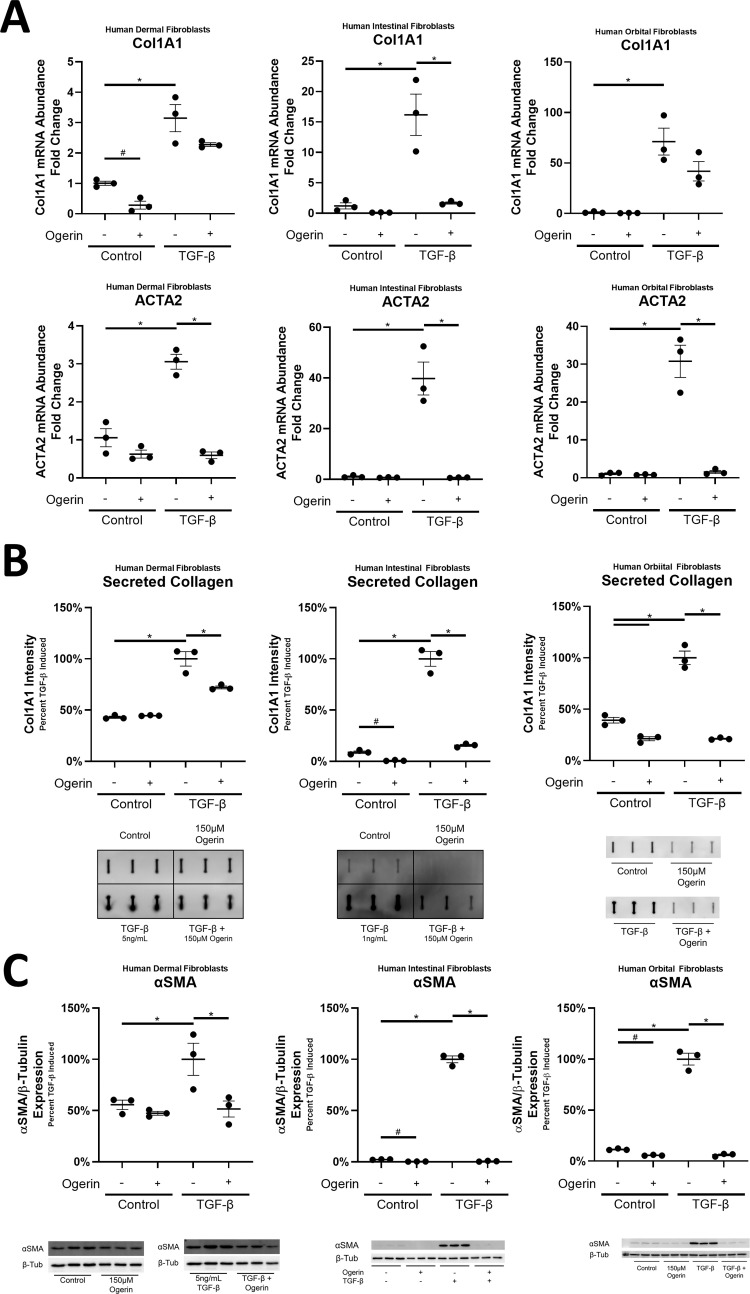
Ogerin inhibits TGF-β induced gene transcription, collagen secretion and myofibroblast differentiation in human dermal, intestinal, and orbital fibroblasts. A: Human Dermal, Intestinal, and Orbital fibroblasts were co-treated with TGF-β (5ng/mL Dermal, 1ng/mL Intestinal and Orbital) and/or 150μM Ogerin for 48 hours. Col1A1 and ACTA2 mRNA transcript levels were analyzed via qRT-PCR. Transcript abundance standardized to 18s rRNA by the ΔΔCt method and expressed as a fold change of control. B: Human Dermal, Intestinal, and Orbital fibroblasts co-treated with TGF-β (5ng/mL Dermal, 1ng/mL Intestinal and Orbital) and/or 150μM Ogerin for 72 hours. Cell culture supernatant analyzed for secreted Col1A1 via slot blot. Band expression was standardized as percent TGF-β induced. C: Human Dermal, Intestinal, and Orbital fibroblasts co-treated with TGF-β (5ng/mL Dermal, 1ng/mL Intestinal and Orbital) and/or 150μM Ogerin for 72 hours. αSMA Band expression was standardized to β-Tubulin, expressed as percent TGF-β induced. n = 3 per cell line per treatment group, dots representative of technical replicates with SEM. * = p < 0.05, by One -Way ANOVA with Tukey’s Post-Hoc Test for Multiple Comparisons.

TGF-β induced myofibroblast differentiation is central to fibrotic diseases throughout the body [108,109]. We utilized fibroblasts from different organ systems to discern if the phenotypic and mechanistic effects of Ogerin were unique to pulmonary fibroblasts, or more broadly applicable. While the magnitude of TGF-β induced myofibroblast differentiation varied across human dermal, intestinal, and orbital fibroblasts, the inhibitory effects of Ogerin previously observed in lung fibroblasts were conserved (Figs 8A–8C and S9). Baseline expression of αSMA and secretion of Col1A1 was comparatively high in dermal fibroblasts and required higher doses (5 ng/mL vs 1 ng/mL) of TGF-β to induce myofibroblast differentiation. The inhibitory effect of 150μM Ogerin on dermal fibroblast were less significant than on intestinal and dermal fibroblast where TGF-β induced αSMA and Collagen secretion were completely inhibited by Ogerin.

**Fig 9:**
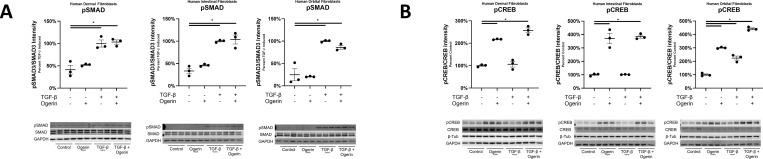
Ogerin does not inhibit TGF-β induced SMAD3 phosphorylation, and induces Gα_s_ signaling in human dermal, intestinal, and orbital fibroblasts. A,B: Human Dermal, Intestinal, and Orbital fibroblasts were co-treated with TGF-β (5ng/mL Dermal, 1ng/mL Intestinal and Orbital) and/or 150μM Ogerin for 40 minutes. Whole cell lysates were analyzed for phosphorylated SMAD3 expression via Western Blot. Phosphorylated SMAD3 band intensity was standardized to total SMAD3 band intensity and expressed as a percent of TGF-β induced. Whole cell lysates were analyzed for phosphorylated CREB expression via Western Blot. Phosphorylated CREB intensity was standardized to total CREB expressed as a percent of control. * = p < 0.05, by One -Way ANOVA with Tukey’s Post-Hoc Test for Multiple Comparisons.

As observed in lung fibroblasts, TGF-β induced SMAD3 phosphorylation was not inhibited by Ogerin in human dermal, intestinal, or orbital fibroblasts (Fig 9A). Induction of Gα_s_ signaling, marked by CREB phosphorylation, was also induced by Ogerin in these fibroblasts (Fig 9B). Therefore, the phenotypic properties of Ogerin on myofibroblast differentiation, and the mechanisms underlying the effect are conserved and more broadly applicable to fibroblasts through the body.

## Discussion

Myofibroblast differentiation is central to the pathophysiology of IPF and other fibrosing diseases. As there are currently limited treatment options, new therapeutic targets are desperately needed to improve outcomes for millions of patients suffering with fibrotic diseases. Our data demonstrate that Ogerin, a small molecule positive allosteric modulator of GPR68, inhibits TGF-β induced myofibroblast differentiation. Due to our findings in fibroblasts derived from diverse human tissues, GPR68 may be a broadly relevant therapeutic target in fibrosis. Further, as significant myofibroblast differentiation has already occurred by the time of clinical presentation of fibrotic disease, the ability to reverse established myofibroblast phenotypes has significant implications to improve patient outcomes.

The magnitude to which a given dose of TGF-β induced pro-fibrotic phenotypes, and the level to which this was inhibited by a given dose varied by organ system and disease state. To determine the cause of this variation, further studies on receptor expression, intracellular signaling intermediates, or metabolic effects are needed. The inhibition not only of TGF-β induced gene transcription, but also basal collagen transcription suggests that the mechanism through which Ogerin inhibits pro-fibrotic phenotypes may be independent of TGF-β signaling. Our data however does not preclude basal collagen transcription regulation by TGF-β. Regulation of basal collagen transcription levels via cell culture sources of TGF-β may be significant. The effect of Ogerin on TGF-β production, latent TGF-β activation or degradation of TGF-β are ongoing. Further, regulation of pro-fibrotic fibroblast phenotypes via other growth factors including Vascular endothelial growth factor (VEGF), Fibroblast growth factors (FGF), or Connective tissue growth factor (CTGF) are important to translate our findings *in vivo*, but are outside the scope of this work.

Due to the inhibition of TGF-β induced gene transcription by Ogerin in both fibroblasts and a SMAD reporter assay, we sought to elucidate the effect of Ogerin on canonical TGF-β induced SMAD signaling. Upon TGF-β induced activation of Type I and Type II TGF-β receptors, receptor-regulated SMAD (R-SMAD) proteins become phosphorylated associate with the common mediator SMAD4, and translocation to the nucleus. In the nucleus, activated SMAD complexes promote or repress gene transcription [7,89,99,100,110–115]. In pulmonary fibroblasts, deposition of scar-forming type I collagen in the fibrotic TGF-β response is largely controlled by the R-SMAD SMAD3 [98].

Pre or co-treatment of Ogerin with TGF-β did not inhibit SMAD3 phosphorylation indicating that Ogerin does not disrupt TGF-β binding to, or the kinase functionality of, its receptor. Further, Ogerin does not inhibit the ability of SMAD3 proteins to translocate to the nucleus and bind chromatin. Therefore, we anticipate that there are other critical mechanisms through which Ogerin inhibits TGFβ induced myofibroblast differentiation.

In general, nuclear localization and chromatin binding of SMAD complexes alone is insufficient to induce transcription [100]. Transcriptional activation of SMAD target genes involves transcriptional complexes of co-activators including CBP/p300 [100,101], YAP/TAZ [116], P/CAF, and the core transcriptional machinery [117]. Thus, the mechanism through which Ogerin inhibits TGF-β induced gene transcription may involve inhibition of these or other coactivators. Further, inhibition of TGF-β induced gene transcription in the absence of inhibitory effects on SMAD signaling in this reporter assay has been attributed to inhibition of TAK1 and P38 [97]. While our work focused on canonical TGF-β induced SMAD signaling, interrogation of these alternative TGF-β signaling pathways and transcriptional mediators will further illuminate mechanisms of myofibroblast differentiation.

Ogerin is a positive allosteric modulator of proton induced GPR68 Gα_s_ signaling [68–71]. While proton mediated activation of GPR68 can couple to multiple canonical GPCR pathways [58,65,118], Ogerin inhibits GPR68 mediated activation of the Gα_q_ pathway and potentiates proton induced activation of the Gα_s_ pathway [68]. Canonical Gα_s_ intracellular signaling is marked by activation of Adenylyl Cyclase (AC), and increases in intracellular cAMP. Elevations in intracellular cAMP exert multiple downstream effector functions including activation of protein kinase A (PKA). cAMP binds to regulatory subunits of PKA releasing the active catalytic subunits to phosphorylate target proteins such as cAMP response element-binding protein (CREB) at serine-133 [72,119]. Our data show that Ogerin activates Gα_s_ signaling in fibroblasts from multiple tissues demonstrated by phosphorylation of CREB in a PKA dependent manner. There is substantial evidence that activation of the Gα_s_ pathway inhibits TGF-β induced pro-fibrotic fibroblast phenotypes in the lung, and other organ systems which has been previously summarized [72,73]. While the cumulative body of evidence supporting Gα_s_ GPCRs as inhibitors of pro-fibrotic fibroblast phenotypes has implicated multiple mechanisms through which this occurs [78,106,120,121], recent work has identified inhibition of YAP/TAZ as an important mediator of these effects [28,79,122–124]. Thus, Ogerin may inhibit TGF-β induced gene transcription via inhibition of YAP/TAZ as has been observed with other Gα_s_ coupled GPCRs.

Although this study is limited to findings in fibroblasts *in vitro*, it is crucial to understand the phenotypes and mechanisms of potential druggable targets in order to accurately interpret *in vivo* findings. Ongoing work investigating the effect of Ogerin in the pulmonary epithelium and the potential of Ogerin to inhibit pulmonary fibrosis *in vivo* will expand our understanding of GPR68 in normal and pathological states. Our work demonstrates that the unique pH sensing properties of GPR68 can be harnessed to increase cell signaling and phenotypic outcomes. In the context of fibrosis, this may provide the opportunity for more targeted therapeutic intervention. Lactic acid is elevated in the lungs of patients with IPF, and TGF-β induced myofibroblast differentiation promotes acidification of the extracellular space through increased expression of LDH5 and export of lactate [53]. Further, active regions of lung fibrosis in the bleomycin model of pulmonary fibrosis are acidified [107]. Ongoing work to untangle the interdependent effects of pH on myofibroblast differentiation, TGF-β activation, GPR68 expression and signaling, and other pH sensing GPCRs [57] furthers our understanding of the pathogenesis of IPF and other fibrotic diseases and may, illuminate new therapeutic targets.

## Conclusions

In conclusion, these studies demonstrate that Ogerin, a small molecule positive allosteric modulator of GPR68, inhibits TGF-β induced myofibroblast differentiation in fibroblasts from multiple organ systems. While Ogerin inhibits of TGF-β induced gene transcription there are no effects on SMAD3 signaling. Ogerin induces Gα_s_ signaling, a well-recognized anti-fibrotic signaling network. Intriguingly, and likely somewhat unique to Ogerin and GPR68, acidic pH potentiates both Gα_s_ signaling and the inhibition of myofibroblast differentiation.

## Supporting information

S1 Fig Ogerin inhibits TGF-β induced myofibroblast differentiation in primary human lung fibroblasts in a dose dependent manner. Primary human lung fibroblasts derived from non-fibrotic or IPF patients were co-treated with 1ng/mL TGF-β and/or 50–150μM Ogerin for 72 hours. Lysates were analyzed for αSMA expression level via Western Blot. αSMA band expression was standardized to β-Tubulin, expressed as percent TGF-β induced. * = p < 0.05 by One-Way ANOVA with Tukey’s Post-Hoc Test for Multiple Comparisons. # = p < 0.05 by T-Test.
(TIF)

S2 Fig Ogerin inhibits TGF-β induced myofibroblast differentiation in primary human lung fibroblasts in a dose dependent manner. Primary human lung fibroblasts (Non-Fibrotic Donor #1) co-treated with 1ng/mL TGF-β and/or 100μM Ogerin for 72 hours. Cells were fixed, permeabilized and stained for αSMA (Green) and nuclei (DAPI, Blue). The top left panel is representative of 400x view, other panels are representative 200x magnification. Technical replicates from independently treated wells.
(TIF)

S3 Fig Ogerin inhibits TGF-β induced collagen production in primary human lung fibroblasts at the transcriptional level. Primary human lung fibroblasts derived from non-fibrotic or IPF patients were co-treated with 1ng/mL TGF-β and/or 50–150μM Ogerin for 72 hours. Cell culture supernatant was analyzed for Col1A1 secretion level via slot blot. Band expression was standardized as percent TGF-β induced. * = p < 0.05 by One-Way ANOVA with Tukey’s Post-Hoc Test for Multiple Comparisons. # = p < 0.05 by T-Test.
(TIF)

S4 Fig Ogerin inhibits TGF-β induced Col1A1 transcription. Primary human lung fibroblasts derived from non-fibrotic or IPF patients were co-treated with 1ng/mL TGF-β and/or 50–150μM Ogerin for 48 hours. Col1A1 RNA transcript levels were analyzed via qRT-PCR. Transcript abundance was standardized to 18s rRNA by the ΔΔCt method and quantified as percent TGF-β induced. * = p < 0.05 by One-Way ANOVA with Tukey’s Post-Hoc Test for Multiple Comparisons. # = p < 0.05 by T-Test.
(TIF)

S5 Fig Ogerin inhibits TGF-β induced Col3A1 Transcription. Primary human lung fibroblasts derived from non-fibrotic or IPF patients were co-treated with 1ng/mL TGF-β and/or 50–150μM Ogerin for 48 hours. Col3a1 RNA transcript levels were analyzed via qRT-PCR. Transcript abundance was standardized to 18s rRNA by the ΔΔCt method and quantified as percent TGF-β induced. * = p < 0.05 by One-Way ANOVA with Tukey’s Post-Hoc Test for Multiple Comparisons. # = p < 0.05 by T-Test.
(TIF)

S6 Fig Representative images of PHLFs. Representative images of primary human lung fibroblasts derived from non-fibrotic or IPF subjects treated +/- DMSO Vehicle, +/- 1ng/mL TGF-β, +/- 50–150μM Ogerin, or 5 μg/mL Puromycin for 72 hours.
(TIF)

S7 Fig Ogerin pre-treatment does not inhibit TGF-β induced SMAD phosphorylation. A: Primary human lung fibroblasts derived from non-fibrotic or IPF patients pre-treated +/-150μM Ogerin for 40 Minutes followed by 1ng/mL TGF-β for 40 minutes. Phosphorylated SMAD3 levels were determined by Western Blot, Phosphorylated SMAD3 intensity was standardized to total SMAD3 expressed as a percent of TGF-β induced. * = p < 0.05 by One-Way ANOVA with Tukey’s Post-Hoc Test for Multiple Comparisons.
(TIF)

S8 Fig Ogerin’s induction of CREB phosphorylation potentiated by acidic pH in CO_2_ independent media. Primary human lung fibroblasts derived from non-fibrotic or IPF patients were treated +/- 100μM Ogerin for 40 Minutes in pH adjusted CO_2_ Independent Media. Whole cell lysates were analyzed for phosphorylated CREB expression via Western Blot. Phosphorylated CREB intensity standardized to total CREB expressed as a percent of control. n = 3 per donor per treatment group, dots representative of technical replicates with SEM.
(TIF)

S9 Fig Representative images of human dermal, intestinal, and orbital fibroblasts. Representative images of human dermal, intestinal, and orbital fibroblasts treated +/- DMSO Vehicle, +/- 1-5ng/mL TGF-β, +/- 150μM Ogerin for 72 hours.
(TIF)

S1 Methods Extended materials and methods. (PDF)

S1 Raw images Original unedited and uncropped blots. (PDF)
