# Supplementary figures and images for: Ogerin mediated inhibition of TGF-β(1) induced myofibroblast differentiation is potentiated by acidic pH
Source: PLoS One. 2022 Jul 28;17(7):e0271608. doi: 10.1371/journal.pone.0271608 (PMC9333254; doi:10.1371/journal.pone.0271608)

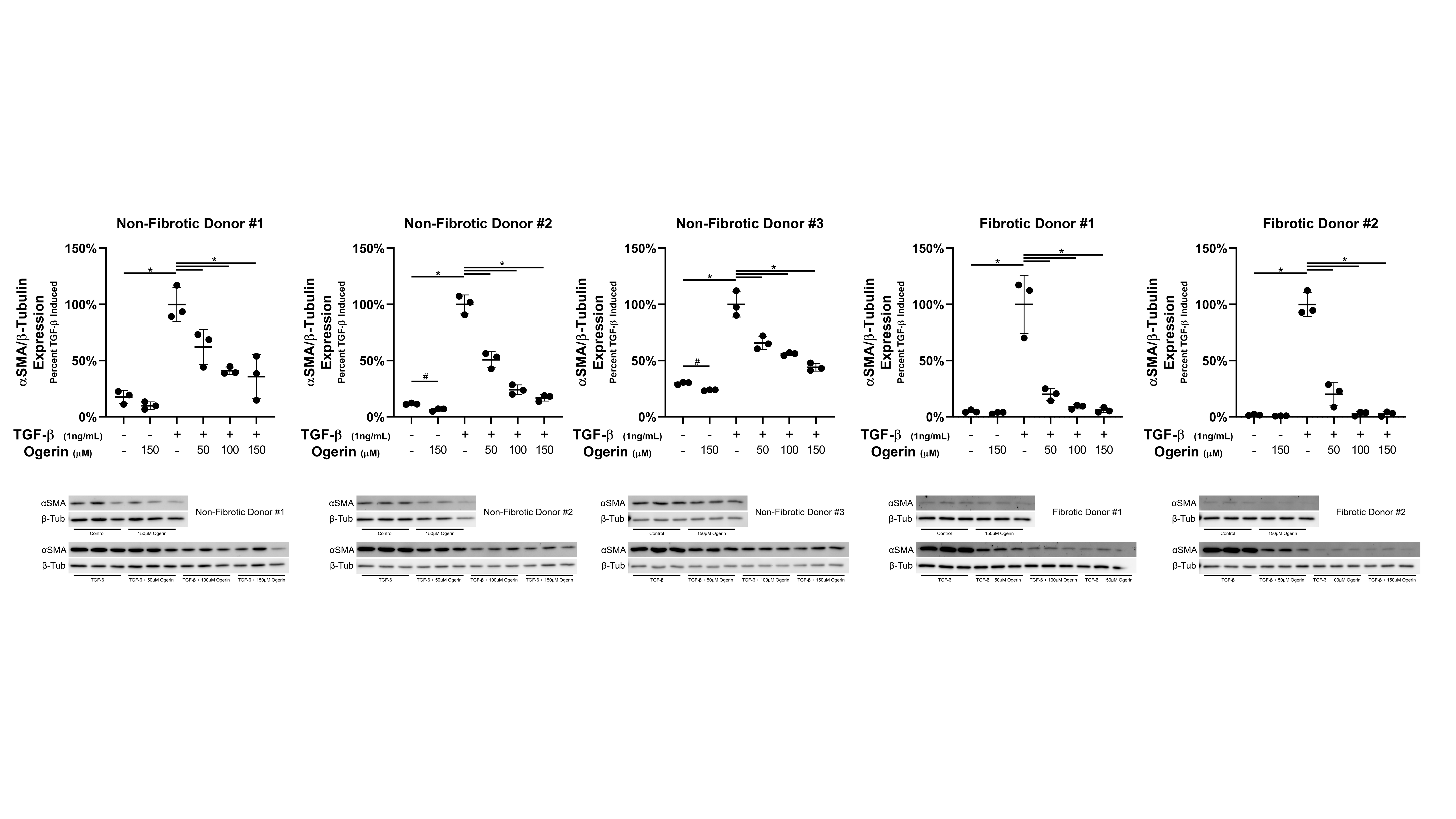

Supplement: S1 Fig — Primary human lung fibroblasts derived from non-fibrotic or IPF patients were co-treated with 1ng/mL TGF-β and/or 50–150μM Ogerin for 72 hours. Lysates were analyzed for αSMA expression level via Western Blot. αSMA band expression was standardized to β-Tubulin, expressed as percent TGF-β induced. * = p < 0.05 by One-Way ANOVA with Tukey’s Post-Hoc Test for Multiple Comparisons. # = p < 0.05 by T-Test. (TIF) [file pone.0271608.s001.tif]

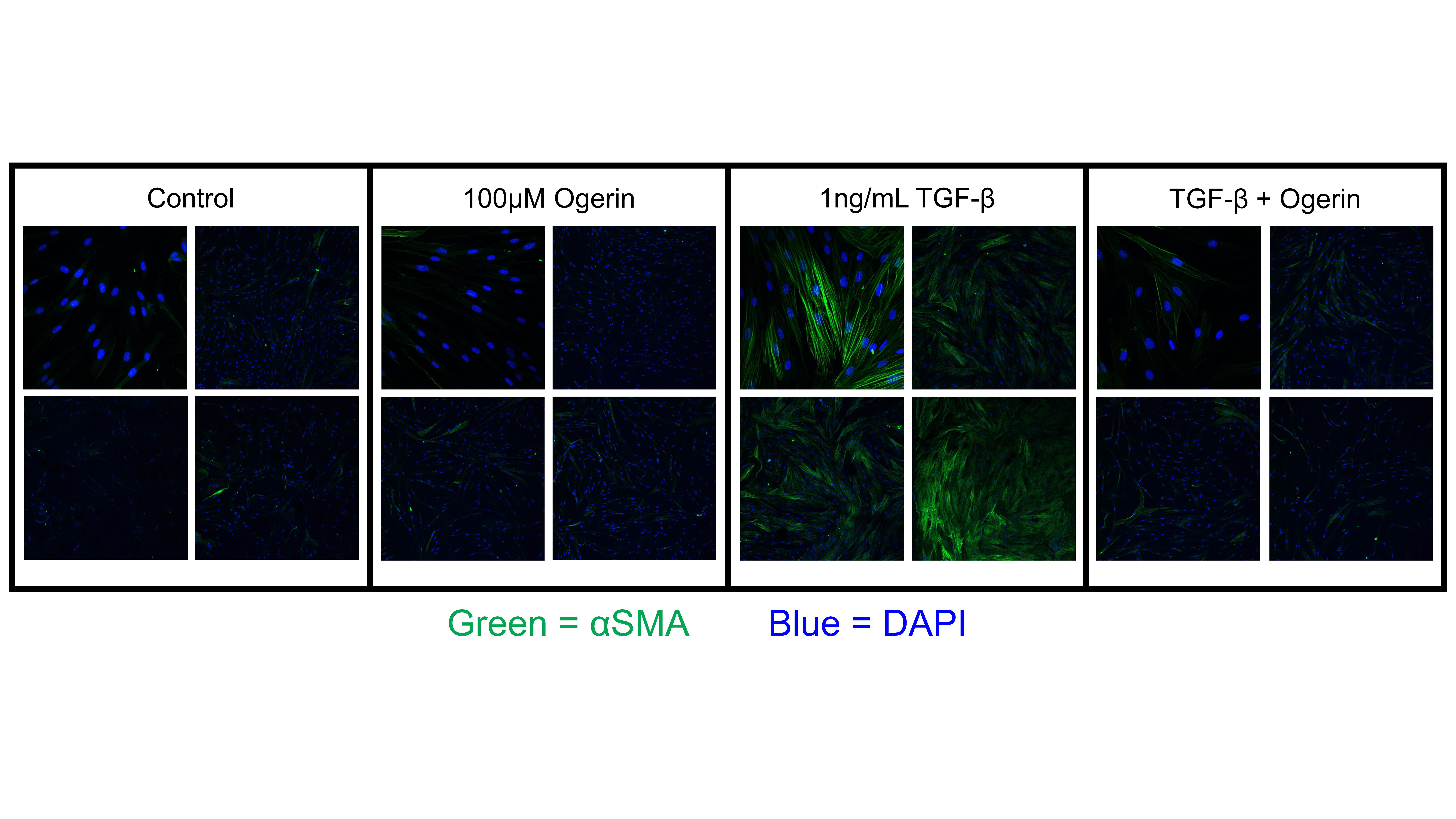

Supplement: S2 Fig — Primary human lung fibroblasts (Non-Fibrotic Donor #1) co-treated with 1ng/mL TGF-β and/or 100μM Ogerin for 72 hours. Cells were fixed, permeabilized and stained for αSMA (Green) and nuclei (DAPI, Blue). The top left panel is representative of 400x view, other panels are representative 200x magnification. Technical replicates from independently treated wells. (TIF) [file pone.0271608.s002.tif]

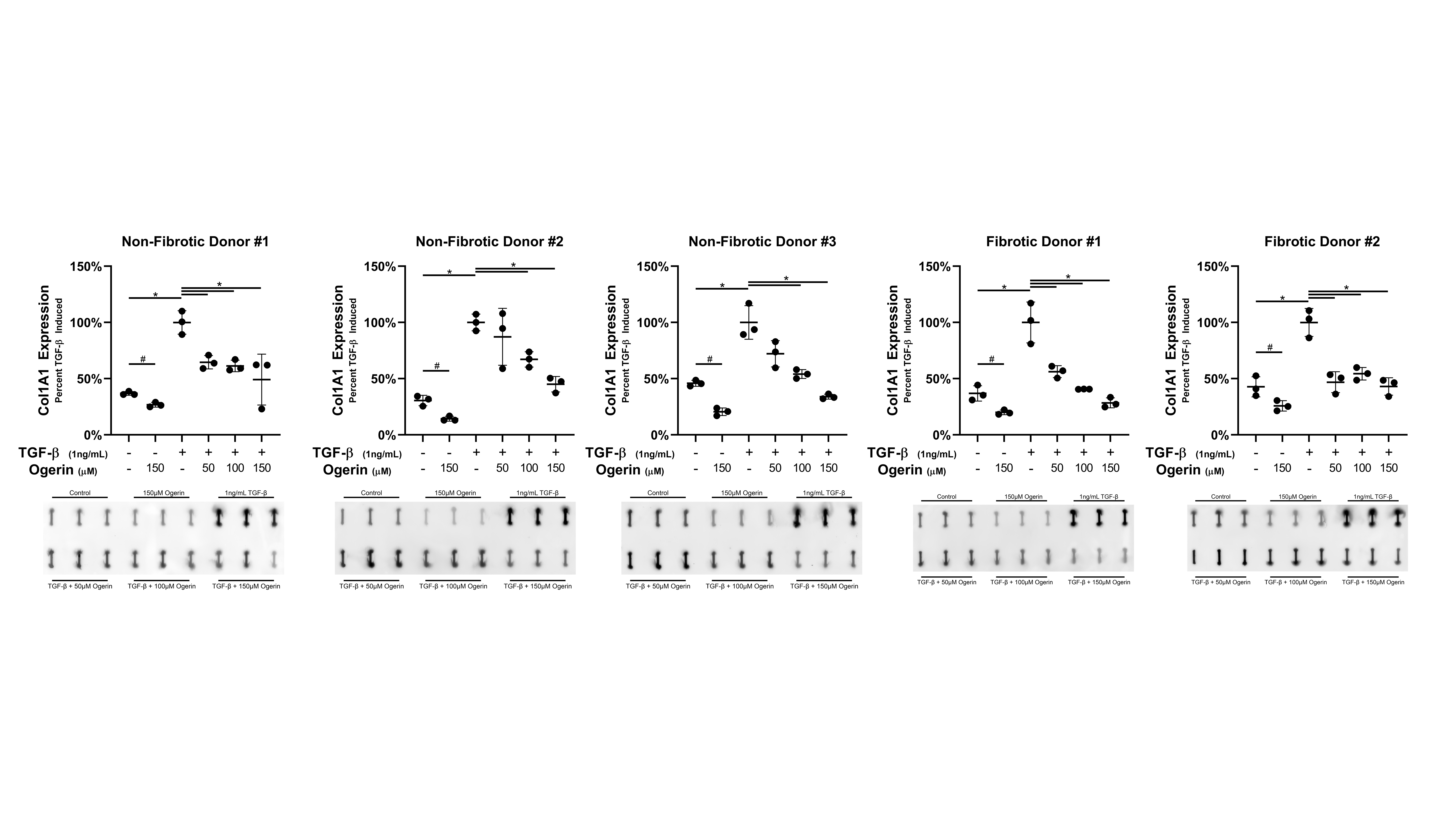

Supplement: S3 Fig — Primary human lung fibroblasts derived from non-fibrotic or IPF patients were co-treated with 1ng/mL TGF-β and/or 50–150μM Ogerin for 72 hours. Cell culture supernatant was analyzed for Col1A1 secretion level via slot blot. Band expression was standardized as percent TGF-β induced. * = p < 0.05 by One-Way ANOVA with Tukey’s Post-Hoc Test for Multiple Comparisons. # = p < 0.05 by T-Test. (TIF) [file pone.0271608.s003.tif]

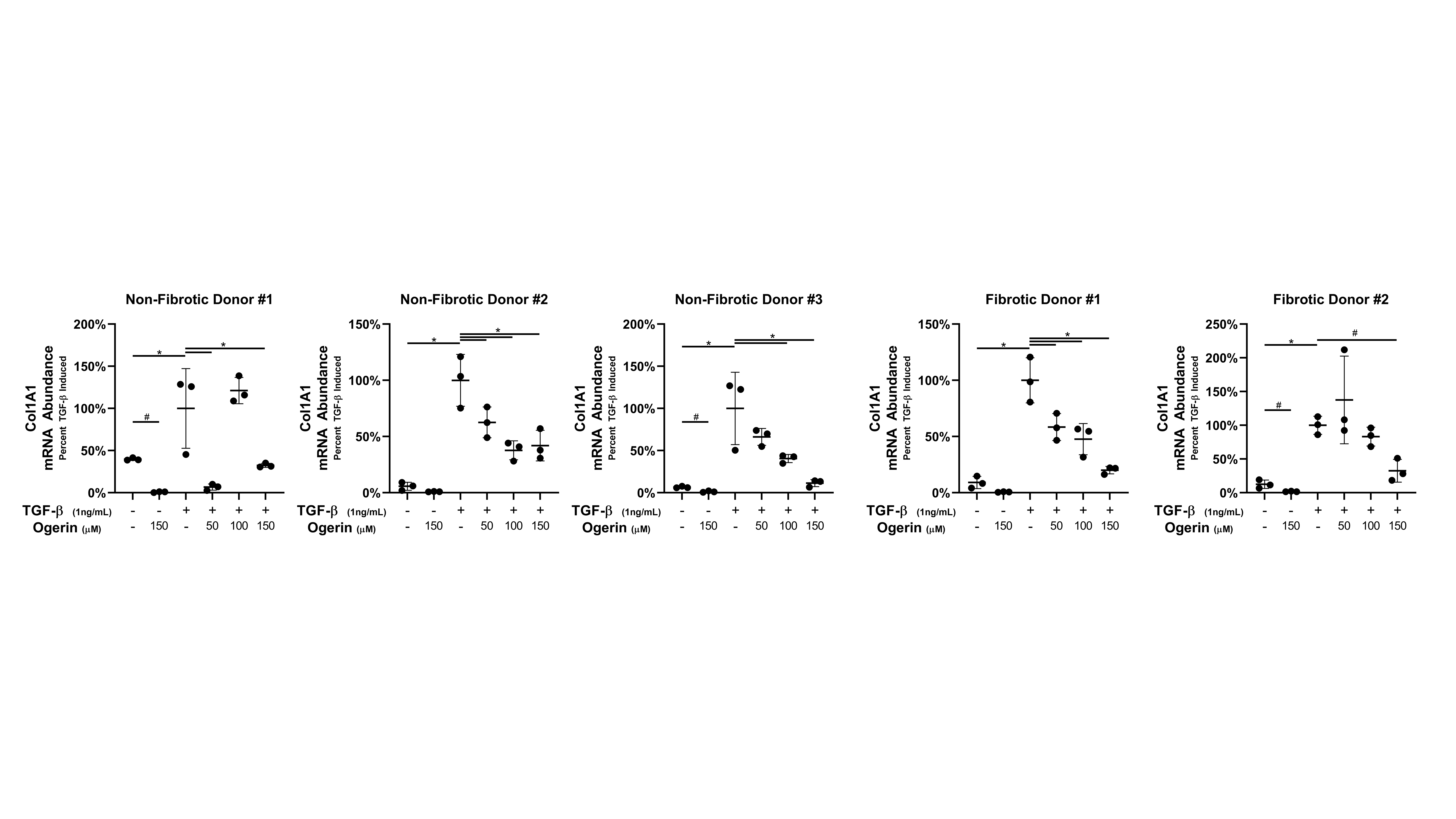

Supplement: S4 Fig — Primary human lung fibroblasts derived from non-fibrotic or IPF patients were co-treated with 1ng/mL TGF-β and/or 50–150μM Ogerin for 48 hours. Col1A1 RNA transcript levels were analyzed via qRT-PCR. Transcript abundance was standardized to 18s rRNA by the ΔΔCt method and quantified as percent TGF-β induced. * = p < 0.05 by One-Way ANOVA with Tukey’s Post-Hoc Test for Multiple Comparisons. # = p < 0.05 by T-Test. (TIF) [file pone.0271608.s004.tif]

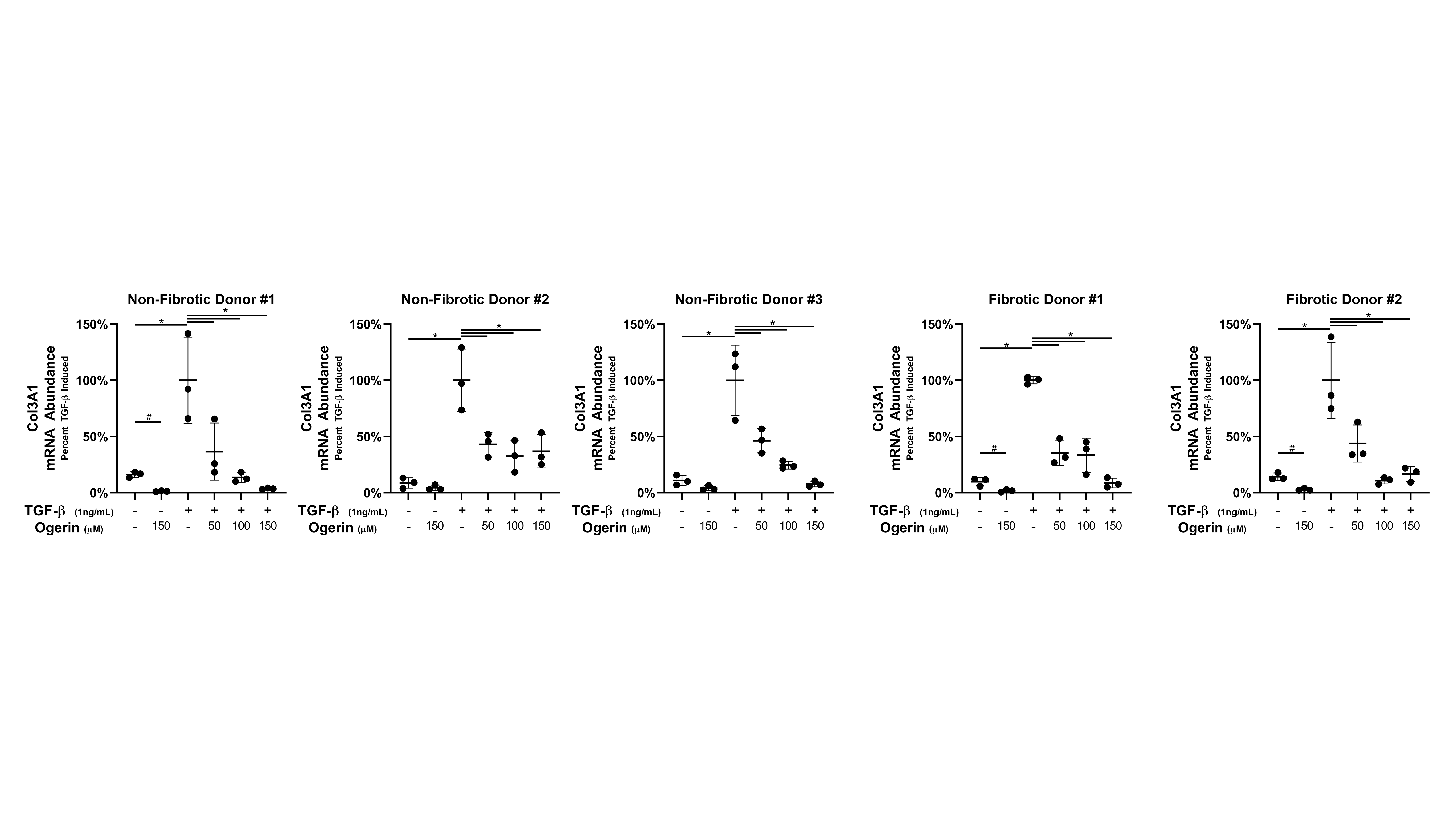

Supplement: S5 Fig — Primary human lung fibroblasts derived from non-fibrotic or IPF patients were co-treated with 1ng/mL TGF-β and/or 50–150μM Ogerin for 48 hours. Col3a1 RNA transcript levels were analyzed via qRT-PCR. Transcript abundance was standardized to 18s rRNA by the ΔΔCt method and quantified as percent TGF-β induced. * = p < 0.05 by One-Way ANOVA with Tukey’s Post-Hoc Test for Multiple Comparisons. # = p < 0.05 by T-Test. (TIF) [file pone.0271608.s005.tif]

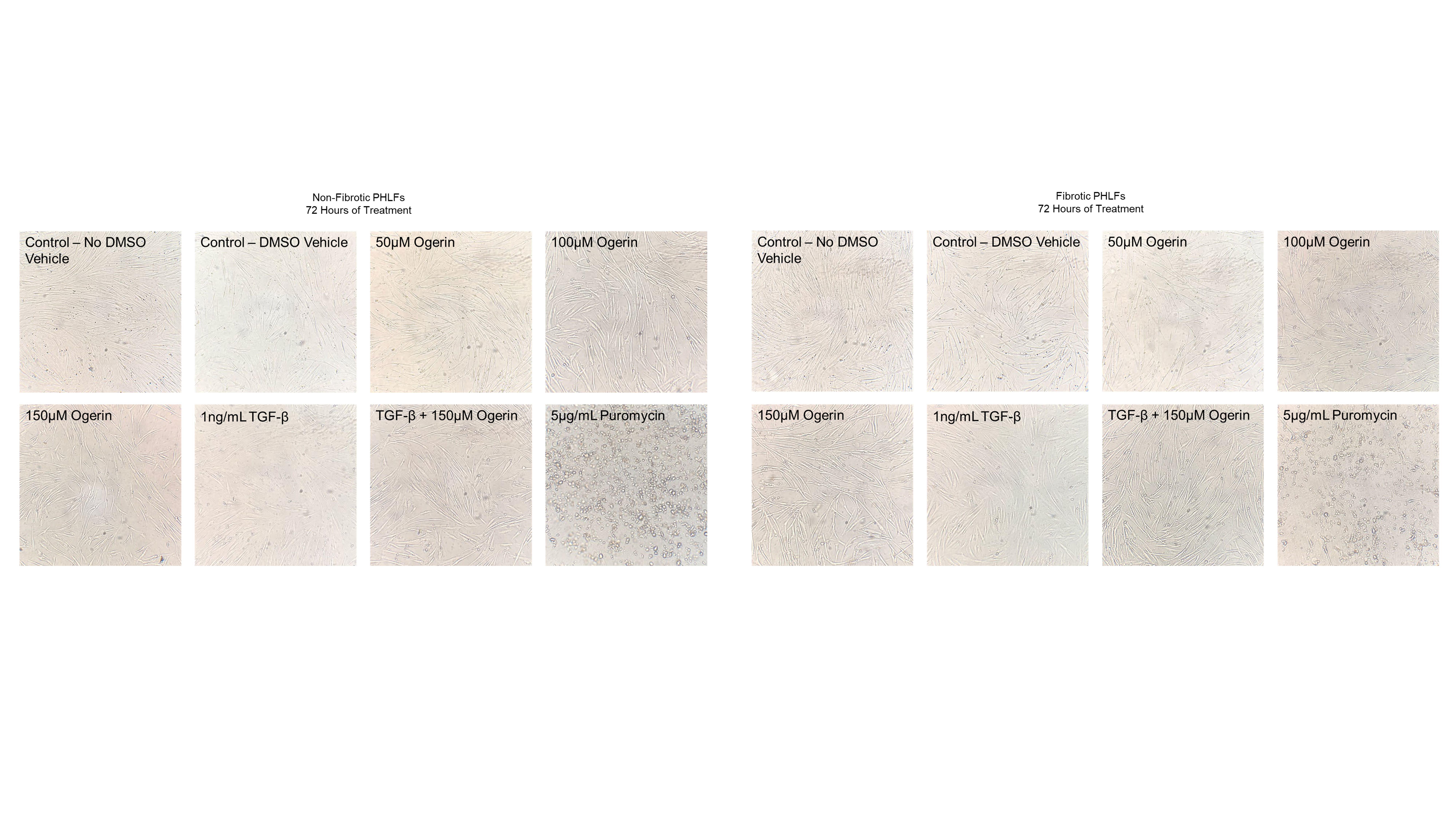

Supplement: S6 Fig — Representative images of primary human lung fibroblasts derived from non-fibrotic or IPF subjects treated +/- DMSO Vehicle, +/- 1ng/mL TGF-β, +/- 50–150μM Ogerin, or 5 μg/mL Puromycin for 72 hours. (TIF) [file pone.0271608.s006.tif]

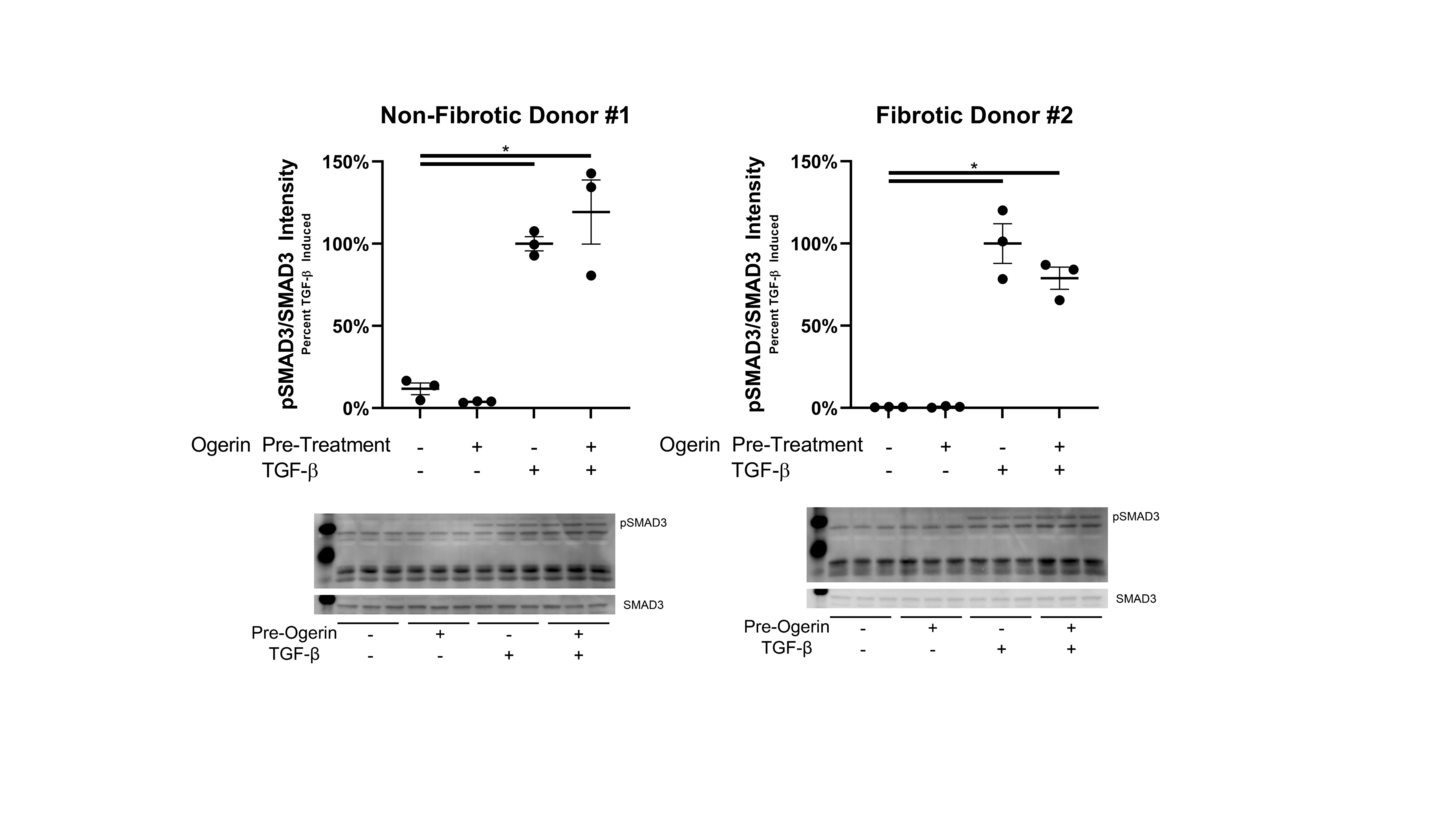

Supplement: S7 Fig — A: Primary human lung fibroblasts derived from non-fibrotic or IPF patients pre-treated +/-150μM Ogerin for 40 Minutes followed by 1ng/mL TGF-β for 40 minutes. Phosphorylated SMAD3 levels were determined by Western Blot, Phosphorylated SMAD3 intensity was standardized to total SMAD3 expressed as a percent of TGF-β induced. * = p < 0.05 by One-Way ANOVA with Tukey’s Post-Hoc Test for Multiple Comparisons. (TIF) [file pone.0271608.s007.tif]

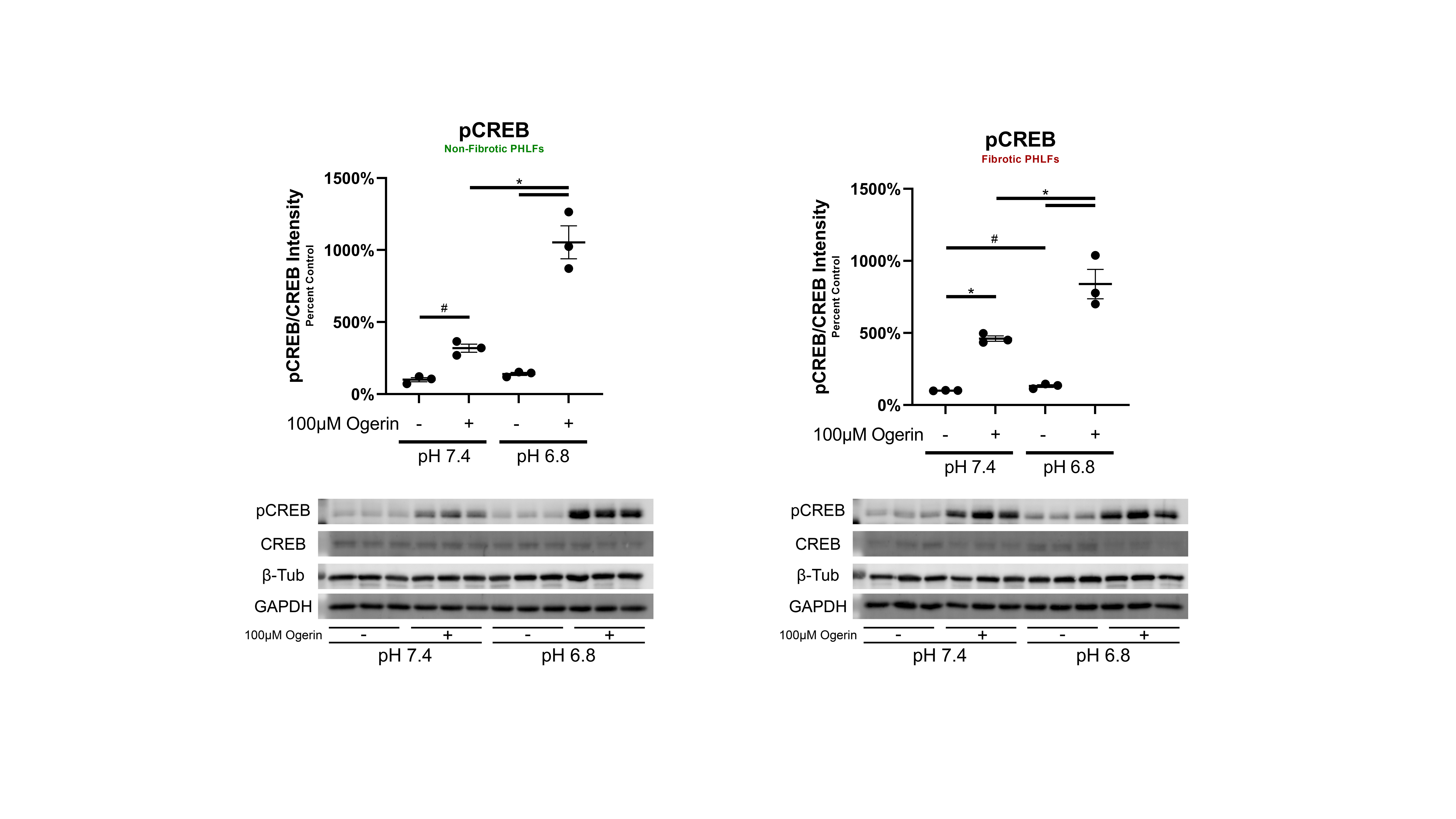

Supplement: S8 Fig — Primary human lung fibroblasts derived from non-fibrotic or IPF patients were treated +/- 100μM Ogerin for 40 Minutes in pH adjusted CO2 Independent Media. Whole cell lysates were analyzed for phosphorylated CREB expression via Western Blot. Phosphorylated CREB intensity standardized to total CREB expressed as a percent of control. n = 3 per donor per treatment group, dots representative of technical replicates with SEM. (TIF) [file pone.0271608.s008.tif]

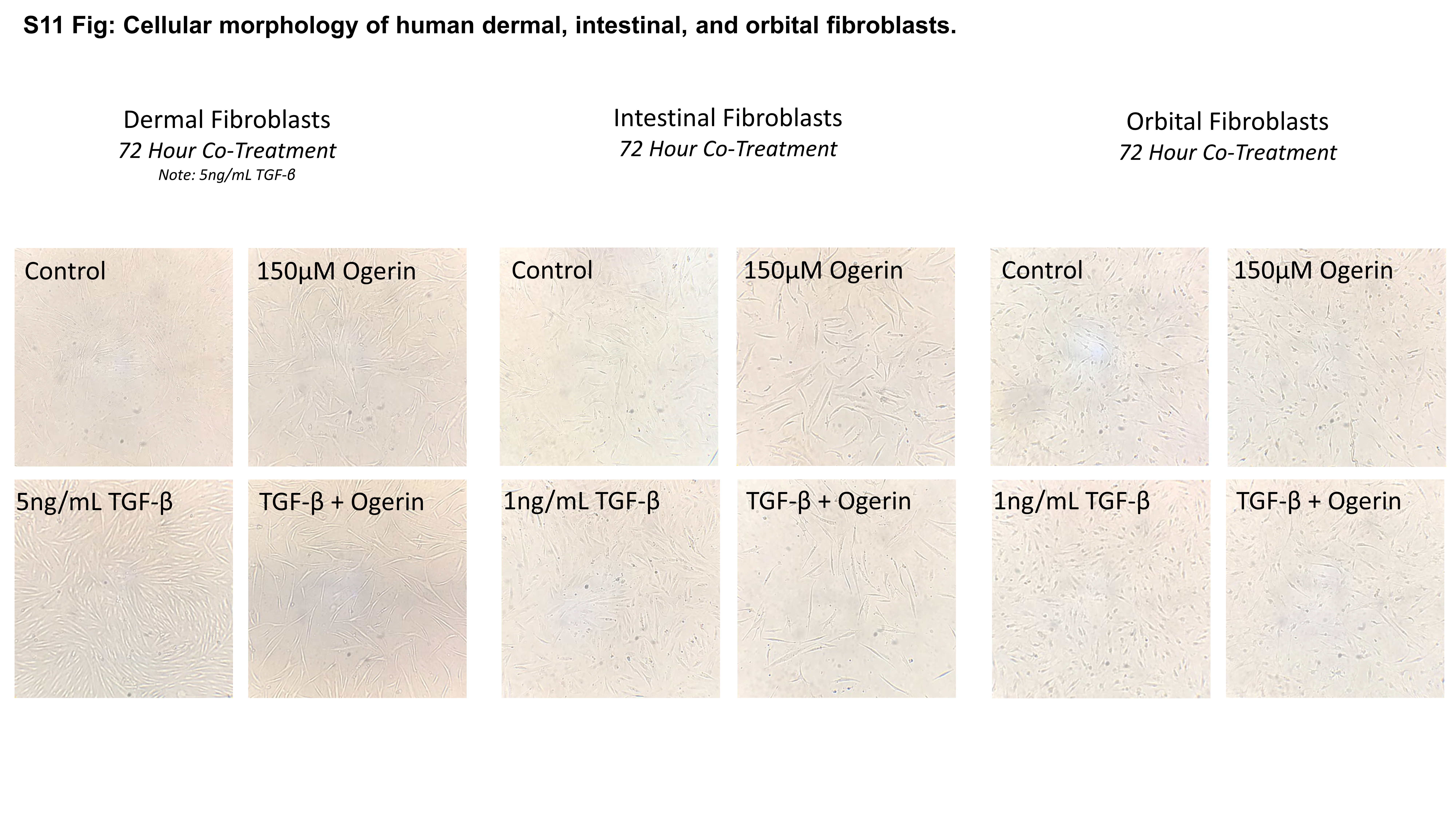

Supplement: S9 Fig — Representative images of human dermal, intestinal, and orbital fibroblasts treated +/- DMSO Vehicle, +/- 1-5ng/mL TGF-β, +/- 150μM Ogerin for 72 hours. (TIF) [file pone.0271608.s009.tif]
