# Supplementary material for: Ogerin mediated inhibition of TGF-β(1) induced myofibroblast differentiation is potentiated by acidic pH
Source: PLoS One. 2022 Jul 28;17(7):e0271608. doi: 10.1371/journal.pone.0271608 (PMC9333254; doi:10.1371/journal.pone.0271608)
Supplement: S1 Methods — (PDF) [file pone.0271608.s010.pdf]

Extended Methods

Cell Culture

Primary Human Lung Fibroblasts (PHLFs) isolated as previously described (1-3) were obtained with informed consent under the approval of the Institutional Review Board of the University of Rochester. Available donor information for cell lines used in this work are as follows:

Primary Human Lung Fibroblasts	Age	Sex	Diagnosis	Smoking History
Non-Fibrotic Donor #1	45	F	Metastatic Melanoma	None
Non-Fibrotic Donor #2	67	F	Respiratory Bronchiolitis with Emphysema	40 pack/years
Non-Fibrotic Donor #3	71	M	Non-Small Cell Carcinoma	56 pack/years
Fibrotic Donor #1	N/A	N/A	IPF	N/A
Fibrotic Donor #2	N/A	N/A	IPF - Severe	N/A

Dermal and orbital fibroblasts were provided by CFW and are previously described (4-6). Low passage (4-8) fibroblasts were revived from liquid nitrogen storage (1 million cells/mL in 90% FBS + 10% dimethyl sulfoxide (DMSO)). Cells were grown in MEM (Gibco™ 11095080) supplemented with 1x Antibiotic-Antimycotic (Gibco™ 15240096), 2mM L-Glutamine (Gibco™ 25030149), and 10% FBS (Corning® Fetal Bovine Serum, 500 mL, Regular, USDA Approved Origin Product Number 35-010-CV, Lot 35010162) at 37°C in 7% CO_2 to ~80% confluency in T175 flasks. Cells were dissociated with 2.5mL Trypsin-EDTA (0.05%) (Gibco™ 25300062), and seeded to experimental plates..

Treatment Preparation

Treatment of cell cultures was performed in MEM as previously described however with a final FBS concentration of 0.5%. All treatment groups received equal concentrations of the Dimethyl Sulfoxide (DMSO) vehicle. For pH adjusted experiments, PBS or CO_2 Independent media (Gibco™ 18045088) supplemented with 0.5% FBS. 1x Antibiotic-Antimycotic, 2mM L-Glutamine was prepared at indicated pHusing microliter volumes of 12N HCL or 10N NaOH. Solutions were allowed to stabilize >24 hours at which time indicated pH was confirmed, and experiments performed in an incubator with room-air levels of CO_2.

Cell Culture Reagents

Ogerin (2-[4-Amino-6-[(phenylmethyl)amino]-1,3,5-triazin-2-yl]-benzenemethanol, [2-[4-Amino-6-(benzylamino)-1,3,5-triazin-2-yl]phenyl]methanol, Sigma-Aldrich SML1482, Source #016M4601V, Batch #0000032886, CAS: 1309198-71-7, MW: 307.35 g/mol, assay ≥98% (HPLC)) was prepared as a 10mM stock in Hybri-Max™ sterile filtered DMSO (Sigma-Aldrich D2650), aliquoted to prevent freeze-thaw cycles, stored at -20°C, and used within 6 months of preparation. Recombinant Human TGF-beta 1 Protein (R&D Systems Catalog #240-B rhTGF-β1) was prepared in a resuspension solution of 4mM HCl + 1mg/mL BSA to a concentration of 5ng/μL. H-89 dihydrochloride (Tocris Biosciences 2910) was prepared as a 20mM stock in DMSO. Forskolin (Sigma-Aldrich F3917) was prepared as a 30mM stock in DMSO.

Protein Lysate Isolation and Western Blotting

Following indicated treatment period, treatment media removed and saved for analysis or aspirated. Cells rinsed with PBS and whole cell lysate harvested in 50µL of lysis buffer (50mM Tris, 2% SDS) with 1:100 Protease Inhibitor Cocktail (Sigma-Aldrich P8340), 1:100 Phosphatase Inhibitor Cocktail (Sigma-Aldrich P0044), and 1:100200mM Phenylmethanesulfonyl fluoride in isopropanol (PMSF, Sigma-Aldrich P7626) with gentle scraping. Subcellular Fractionation performed with the Thermo Scientific Subcellular Protein Fractionation Kit for Cultured Cells (Thermo Fisher Scientific 78840) as per the manufacturer's instructions. Protein concentrations determined with Pierce™ BCA Protein Assay (Thermo Scientific 23228). In brief, 10µL of lysate or 2.0mg/mL-0.0mg/mL serially diluted Albumin Standards (Thermo Scientific 23209) and 10µg of protein was prepared with 4x Laemmli Sample Buffer (Bio-Rad 1610747) with 2-Mercaptoethanol and boiled in a water bath for 10 minutes. Gel electrophoresis was performed with 15 well, 1.5mm handcast 10% sodium dodecyl sulfate polyacrylamide stacking gels at 100V (Mini-PROTEAN® Tetra Vertical Electrophoresis Cell, Bio-Rad 1658006FC) with Spectra™ Multicolor Broad Range Protein Ladder (Thermo Scientific™ 26623). Samples were transferred to low fluorescence PVDF membranes (Bio-Rad 1704275) using a Trans-Blot® Turbo™ Transfer System (Bio-Rad 1704150) as per manufacturer's protocols. After transfer, membrane was briefly rinsed with wash buffer (Phosphate Buffered Saline with 0.05% TWEEN® 20 (Sigma-Aldrich P7949) (PBS-T) or Tris Buffered Saline with 0.05% TWEEN® 20 (TBS-T)) as indicated for the primary antibody described below and stored in opaque western blot boxes. Blots were blocked for 1 hour with gentle rocking utilizing the blocking buffer indicated for the primary antibody. Primary antibody was applied as indicated below overnight with gentle rocking at 4°C. Blots were washed 3 times for 5 minutes, and secondary antibody applied for 1 hour with gentle rocking. Blots were again washed 3 times for 5 minutes and imaged on a ChemiDoc MP (Bio-Rad 12003154) system using Auto-Optimal exposure to bands of interest to prevent overexposure.

Slot Blot

Cell media supernatant harvested at indicated time points was stored at -80°C until assayed. 200µL of the supernatant was combined with 300µL PBS and applied to a Low-Fluorescent PVDF membrane that had been activated with 100% Ethanol and rinsed with PBS-T wash buffer using a Harvard Apparatus SHM-24 24-Well Slot Blot Hybridization Manifold. Vacuum was applied for 5 minutes after which blot were rinsed with PBS-T wash buffer, blocked for 1 hour with 5% Milk in PBS-T, incubated overnight at 4°C with gentle rocking using the Col1A1 antibody as described below. Blots were washed 3 times for 5 minutes, and secondary antibody applied for 1 hour with gentle rocking. Blots were again washed 3 times for 5 minutes and imaged on a ChemiDoc MP (Bio-Rad 12003154) system using Auto-Optimal exposure to prevent overexposure.

Primary Antibodies

Target Abbreviation	Antibody Target Protein	Manufacturer	Product ID	Dilution	Buffer	Secondary
αSMA	α-Smooth Muscle Actin	Sigma-Aldrich	A2547	1:10K	5% Milk in PBS-T	520
β-Tub	beta Tubulin	Abcam	ab6046	1:10K	5% Milk in PBS-T	700
Col1A1	Collagen 1A1	Aviva Systems Biology	ARP59999_P050	1:1K	5% Milk in PBS-T	700
pSMAD	Phospho-SMAD3 Ser423/425	Cell Signaling Technology	9520	1:1K	5% BSA in TBS-T	700
SMAD3	SMAD2/3	Cell Signaling Technology	8685	1:1K	5% BSA in TBS-T	700
pCREB	Phospho-CREB Ser133	Cell Signaling Technology	9198	1:1K	5% BSA in TBS-T	700
CREB	Total CREB	Cell Signaling Technology	9104	1:1K	5% BSA in TBS-T	520
GAPDH	GAPDH	Abcam	ab8245	1:10K	5% BSA in TBS-T	520
PARP	Cleaved PARP (Asp214)	Cell Signaling Technology	9541	1:1K	5% BSA in TBS-T	700
HSP90	HSP90	Cell Signaling Technology	4874	1:1K	5% BSA in TBS-T	700
AIF	AIF	Cell Signaling Technology	4642	1:1K	5% BSA in TBS-T	700
Lamin	Lamin A/C	Cell Signaling Technology	4777	1:1K	5% BSA in TBS-T	520

Secondary Antibodies

Antibody Abbreviation	Target Species	Manufacturer	Product Name	Protect ID	Dilution	Buffer
520	Goat Anti-Mouse IgG	Bio-Rad	Starbright™ Blue 520	12005866	1:10K	As Indicated for Primary
700	Goat Anti-Rabbit IgG	Bio-Rad	StarBright™ Blue 700	12004161	1:10K	As Indicated for Primary

Image Lab files were exported and analyzed with Image Lab 6.1 Software for Windows (Bio-Rad 12012931). The channel of interest (auto scaled imaged transformation, no to very minimal user input) was cropped to show target bands (original uncropped blots provided in S1), exported as a TIF file, and composite representations prepared with Adobe Photoshop Elements 2019. Band intensity was determined with Image Lab 6.1 Software, and adjusted volume (band intensity adjusted for background) exported to Microsoft Excel. Band intensity was standardized to control (see Y axis of each figure) by dividing the adjusted volume of interest by the adjusted volume of the control and expressed as a percentage of control or TGF-β induced (see Y axis of each figure).

Immunofluorescence

Primary Human Lung Fibroblasts were grown as previously described and 5,000 cells per well were seeded to 8 well chamber slides (ThermoFisher Scientific 177402PK) and treated as previously

described. Following treatment period, media was removed from wells, cells were rinsed with ice cold PBS, and fixed with ice cold 100% Methanol. Cells were permeabilized with 0.3% Triton™ X-100 (Sigma-Aldrich X100) for 30 minutes and washed 3 times with PBS. Non-specific binding was blocked with a 1-hour incubation of 3% BSA (Millipore-Sigma 2930) + 1% goat serum (Southern Biotech OB006001). αSMA antibody was applied at 1:200 dilution overnight at 4°C in previously described blocking reagent. Cells were washed 3 times with PBS and fluorescent secondary antibody (Alexa Flour 488 Thermo Fisher Scientific A-11017) for 1 hour protected from light. Cells were washed 3 times with PBS and glass coverslips (VWR 48393 059) with ProLong™ Dimond Antifade Mountant with DAPI (Invitrogen P36962). Representative images of 3 separate wells at 200x for each treatment condition, and 1 well per treatment condition at 400x, were captured on an Olympus FV1000 laser scanning confocal microscope with no changes in light intensity or image capture parameters between images. Images were exported and prepared with FIJI (ImageJ) and compiled in Microsoft PowerPoint.

<u>PCR</u>

Following indicated treatment periods, treatment media was removed and cells rinsed with PBS. RNA was isolated using the pellet method. QIAzol™ Lysis Reagent (Qiagen 79306) was added to wells, extract was phase separated following the addition of chloroform, and RNA precipitated with Isopropanol. RNA pellets were washed with 75% EtOH, air dried, and resuspended in H_2O. RNA concentrations were determined with a NanoDrop™ OneC Microvolume UV-Vis Spectrophotometer (Thermo Scientific™). cDNA was generated with 100ng of RNA using iScript™ cDNA Synthesis Kit (Bio-rad 1708890) and thermocycled as per manufacturer's protocol. Resultant cDNA was diluted 1:5 and 2μL utilized for subsequent qPCR applications. iQ™ SYBR® Green Supermix (Bio-rad 1708880) was used as per the manufacturer's instructions and cycled as follows on a Bio-Rad CFX96™ Real-Time System C1000 Touch™ Thermal Cycler: Priming (3 Minutes at 95°C), Denaturation (30 Seconds at 95°C), Elongation (30 Seconds at 58°C), Measure, Cycle, Melt Curve. C_q (or referred to as C_t) values were extracted using CFX Maestro software, and transcripts of interest were quantified using the ΔΔCt method (7) In short, the data transformation is as follows:

Ct Value → ΔCt (Sample - 18S) → ΔΔCt (ΔCt- Untreated Average ΔCt) → Fold Change (2^-(ΔΔCt))
→ Log2 Fold Change (-)ΔΔCt

Standardized fold changes were then converted to percentage. Primers used in this work are described below.

Primer	Direction	Manufacturer	Sequence or Product ID
18s	Forward	IDT	5'- GGT CGC TCG CTC CTC TCC CA -3'
18s	Reverse	IDT	5'- AGG GGC TGA CCG GGT TGG TT -3'
Col3A1	Forward	IDT	5'- TTG AAG GAG GAT GTT CCC ATC T -3'
Col3A1	Reverse	IDT	5'- ACA GAC ACA TAT TTG GCA TGG TT -3'
Col1A1	Forward + Reverse	Bio-Rad	PrimePCR SYBR Green Assay: Col1A1, Human. Unique Assay ID: qHsaCED0002181

Alamar Blue Assay

Cytotoxicity of Ogerin was measured as described previously (8). Primary Human Lung Fibroblasts were grown as previously described and 5,000 cells per well were seeded to 96 well plates (Corning Incorporated Costar® 96 well Assay Plate, White Plate, Clear Bottom with Lid, Tissue Culture Treated) in 50µL of 0.5% FBS MEM. 24 hours after seeding, 50µL of 2x treatment concentration prepped media was added to the wells and allowed to incubate for 72 hours. Following incubation period, 10µL of alamarBlue™ Cell Viability Reagent (Invitrogen™ DAL1100) was added to each well and allowed to incubate for 4 hours. Fluorescence was measured on a Tecan Infinite F Plex microplate reader using Magellan™ V7.2 software with an excitation wavelength of 535 nanometers and emission wavelength of 595 nanometers with 25 flash reads, optimal gain, and a 20µs integration time. Fluorescence intensity values were exported to Microsoft Excel, standardized to DMSO Vehicle Control values and expressed as a percentage.

SMAD Reporter Assay

A previously described TGF-β responsive SMAD reporter cell line was utilized to determine the effect of Ogerin on TGF-β induced gene transcription (9). HEK-293FT cells were stably transfected with a minimal thymidine kinase promotor with 4 tandem SMAD binding elements (SBEs) upstream of the luc2P (luciferase,pGL4.15[luc2P/Hygro] Vector, Promega) gene and selected for hygromycin resistance and TGF-β responsiveness. Cells were grown as previously described in 10% FBS MEM. 50K cells/well were seeded to 96 well plates (Corning Incorporated Costar® 96 well Assay Plate, White Plate, Clear Bottom with Lid, Tissue Culture Treated) in 50µL of 0.5% FBS MEM. 24 hours after seeding, 50µL of 2x treatment concentration (+/- 1-10ng/mL TGF-β, +/- 50-150µM Ogerin) prepped media was added to the wells and allowed to incubate for 24 hours. After incubation period, 100µL of steadylite plus Reporter Gene Assay System (PerkinElmer®) prepared as per manufacturers protocols were added to wells. Plate was mixed at 500RPM on an IKA® MS 3 digital plate shaker protected from light for 10 minutes. Luminescence intensity was measured Tecan Infinite F Plex microplate reader using Magellan™ V7.2 software with no attenuation and a 5000ms integration time. Luminescence intensity values were exported to Microsoft Excel, standardized to DMSO Vehicle Control or TGF-β induced values and expressed as a percentage.

Statistical Analysis and Graphing

All data expressed as indicated on plots with individual technical replicates with range or means +/- SEM. Statistical analysis performed using GraphPad Prism 9 software for Windows (GraphPad Software, San Diego, California USA, www.graphpad.com). $p < 0.05$ considered significant for ordinary one-way ANOVA with Tukey's post hoc test for multiple comparisons, T-tests, and 2-Way ANOVAs with multiple comparisons.

1. Baglole CJ, Reddy SY, Pollock SJ, Feldon SE, Sime PJ, Smith TJ, et al. Isolation and phenotypic characterization of lung fibroblasts. Methods Mol Med. 2005;117:115-27.
2. Jeon KI, Kulkarni A, Woeller CF, Phipps RP, Sime PJ, Hindman HB, et al. Inhibitory effects of PPARgamma ligands on TGF-beta1-induced corneal myofibroblast transformation. Am J Pathol. 2014;184(5):1429-45.
3. Kottmann RM, Kulkarni AA, Smolnycki KA, Lyda E, Dahanayake T, Salibi R, et al. Lactic acid is elevated in idiopathic pulmonary fibrosis and induces myofibroblast differentiation via pH-dependent activation of transforming growth factor-β. American journal of respiratory and critical care medicine. 2012;186(8):740-51.
4. Roztocil E, Hammond CL, Gonzalez MO, Feldon SE, Woeller CF. The aryl hydrocarbon receptor pathway controls matrix metalloproteinase-1 and collagen levels in human orbital fibroblasts. Sci Rep. 2020;10(1):8477.
5. Guo N, Woeller CF, Feldon SE, Phipps RP. Peroxisome proliferator-activated receptor gamma ligands inhibit transforming growth factor-beta-induced, hyaluronan-dependent, T cell adhesion to orbital fibroblasts. The Journal of biological chemistry. 2011;286(21):18856-67.
6. Fedyk ER, Jones D, Critchley HO, Phipps RP, Blieden TM, Springer TA. Expression of stromal-derived factor-1 is decreased by IL-1 and TNF and in dermal wound healing. J Immunol. 2001;166(9):5749-54.
7. Livak KJ, Schmittgen TD. Analysis of relative gene expression data using real-time quantitative PCR and the 2(-Delta Delta C(T)) Method. Methods. 2001;25(4):402-8.
8. Lancaster Michael V, Fields Rebecca D, inventors; ALAMAR BIOSCIENCES LAB INC, assignee. Antibiotic and cytotoxic drug susceptibility assays using resazurin and poising agents1995 1995/02/28/Application date.
9. Woeller CF, O'Loughlin CW, Roztocil E, Feldon SE, Phipps RP. Salinomycin and other polyether ionophores are a new class of antiscarring agent. The Journal of biological chemistry. 2015;290(6):3563-75.
