# Supplementary material for: Ogerin mediated inhibition of TGF-β(1) induced myofibroblast differentiation is potentiated by acidic pH
Source: PLoS One. 2022 Jul 28;17(7):e0271608. doi: 10.1371/journal.pone.0271608 (PMC9333254; doi:10.1371/journal.pone.0271608)
Supplement: S1 Raw images — (PDF) [file pone.0271608.s011.pdf]

Original Uncropped Blots

Ogerin Mediated Inhibition of TGF-β(1) Induced Myofibroblast Differentiation is Potentiated by Acidic pH

Tyler J. Bell, David J. Nagel, Collynn F. Woeller, R. Mathew Kottmann

S3 Figure

Non-Fibrotic Donor #1

αSMA

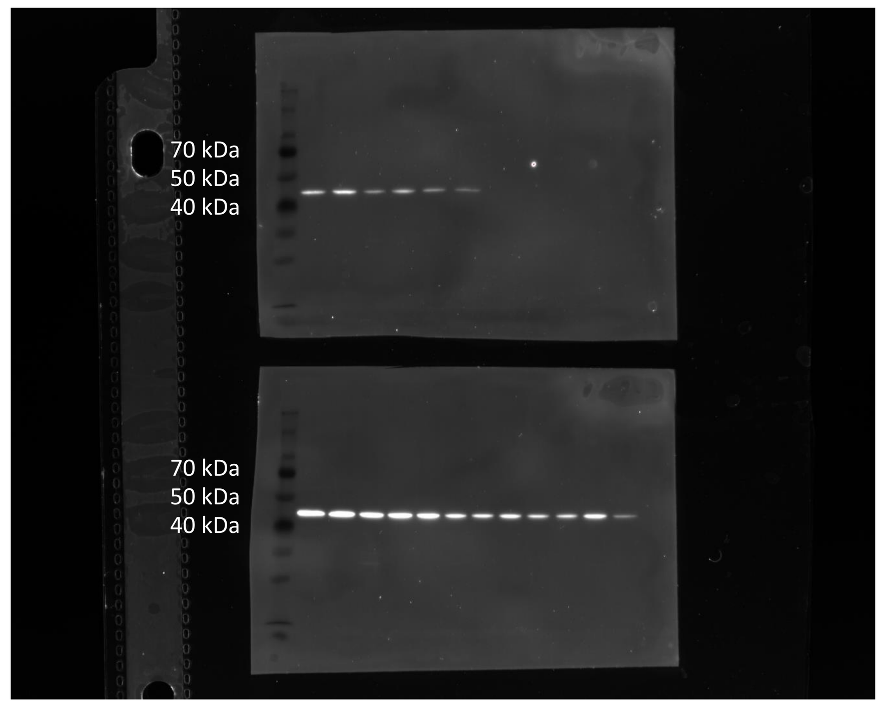

β-Tub

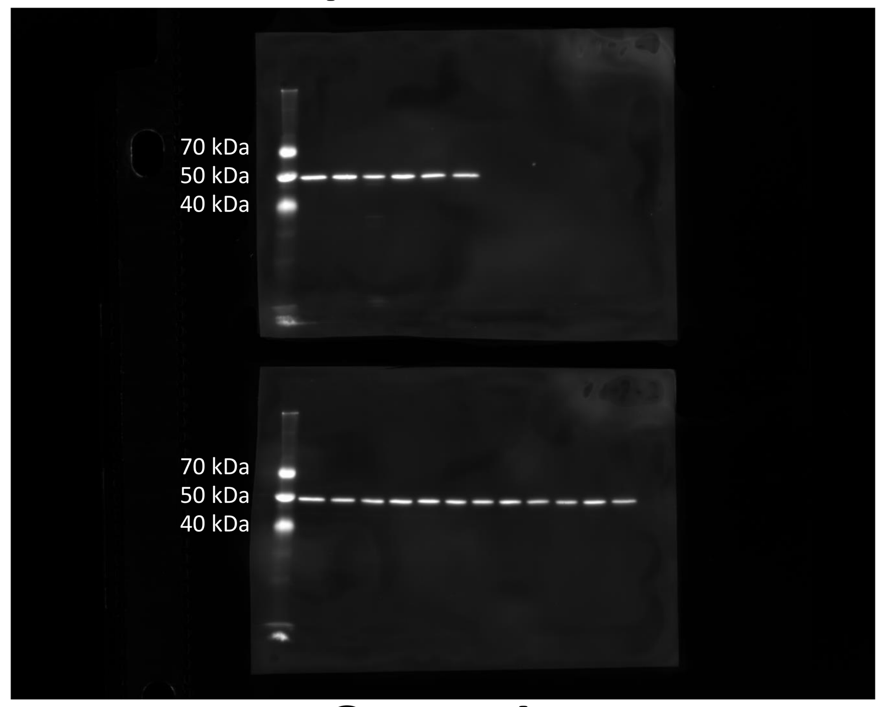

Non-Fibrotic Donor #2

αSMA

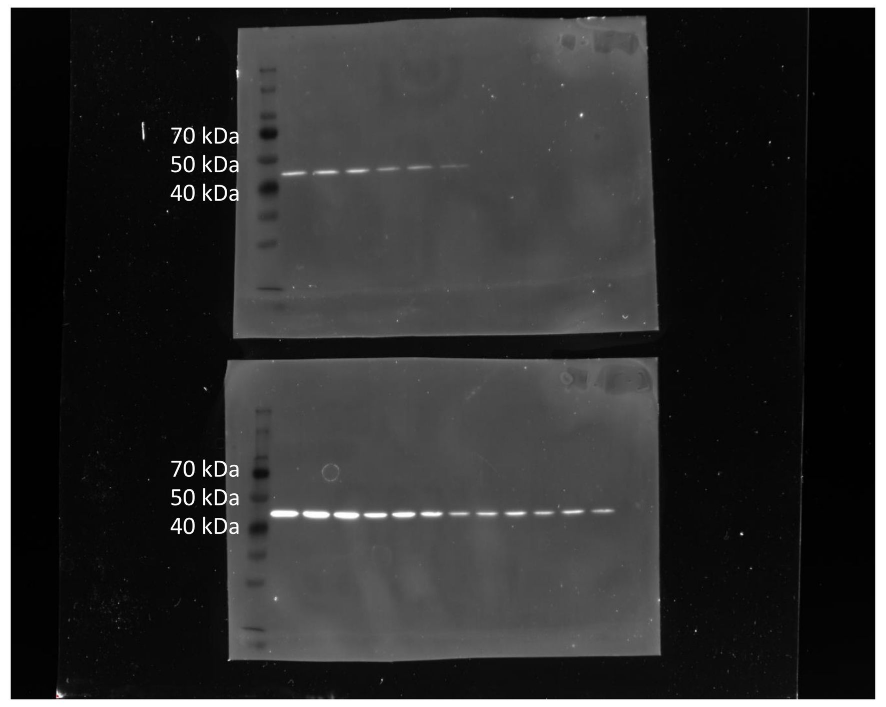

β-Tub

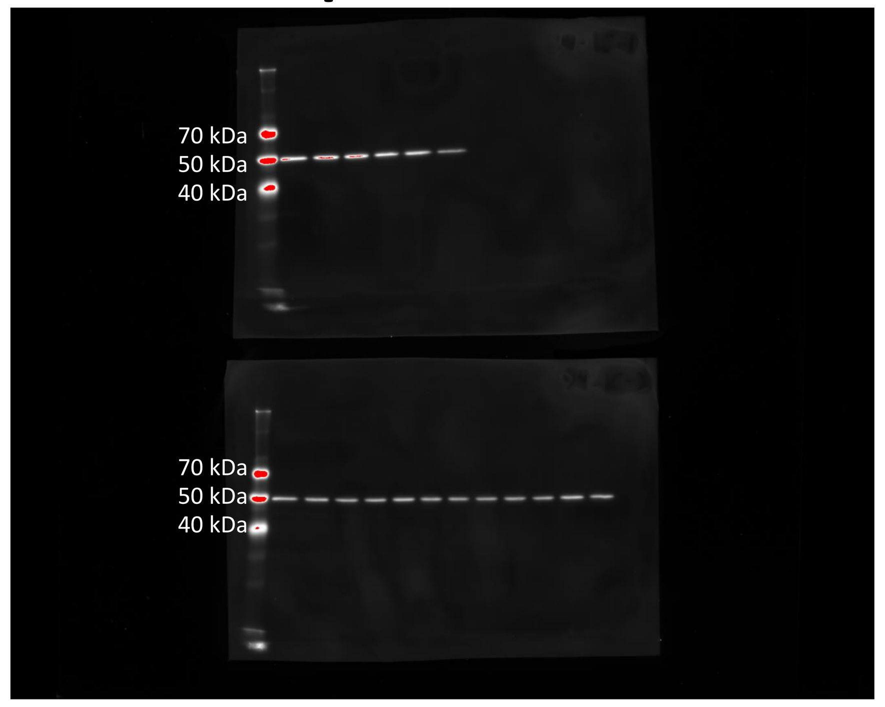

Non-Fibrotic Donor #3

αSMA

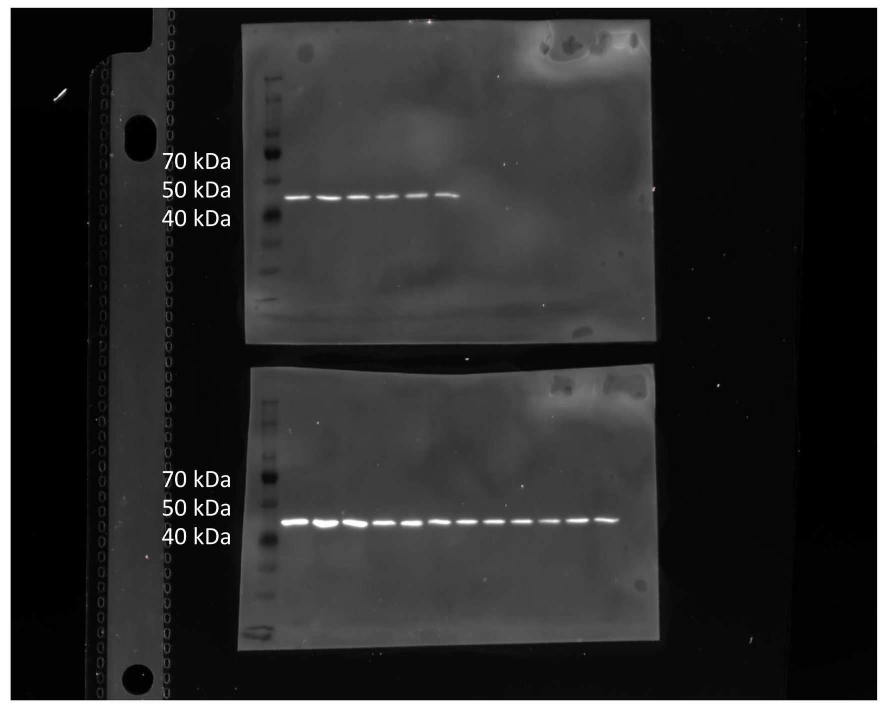

β-Tub

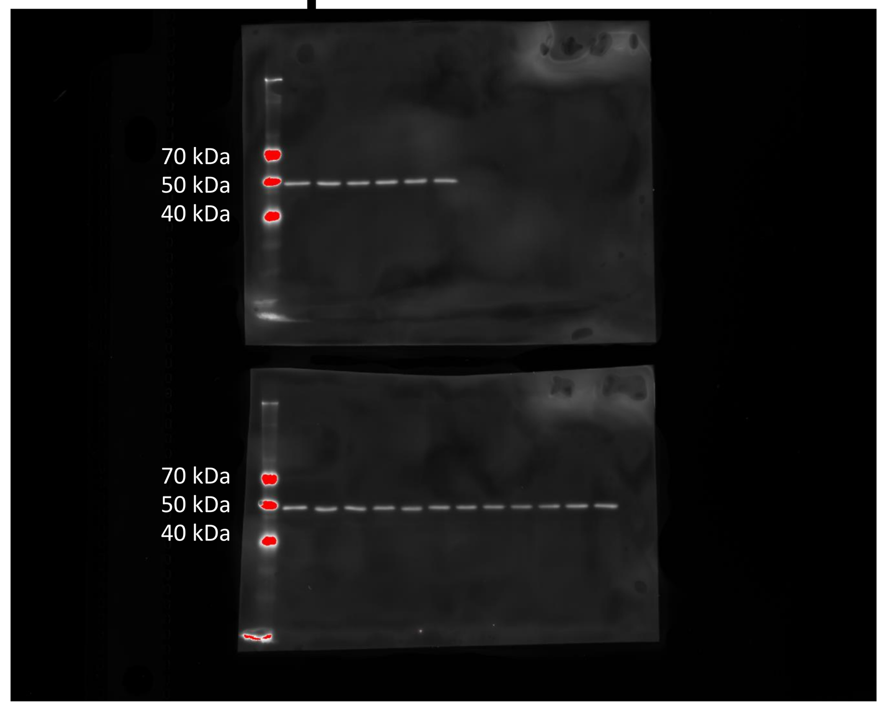

Layout for All Blots

X X X — Control

X X X — 150 µM Ogerin

X X X — 1 ng/mL TGF-β

X X X — TGF-β + 50 µM Ogerin

X X X — TGF-β + 100 µM Ogerin

X X X — TGF-β + 150 µM Ogerin

Fibrotic Donor #1

αSMA

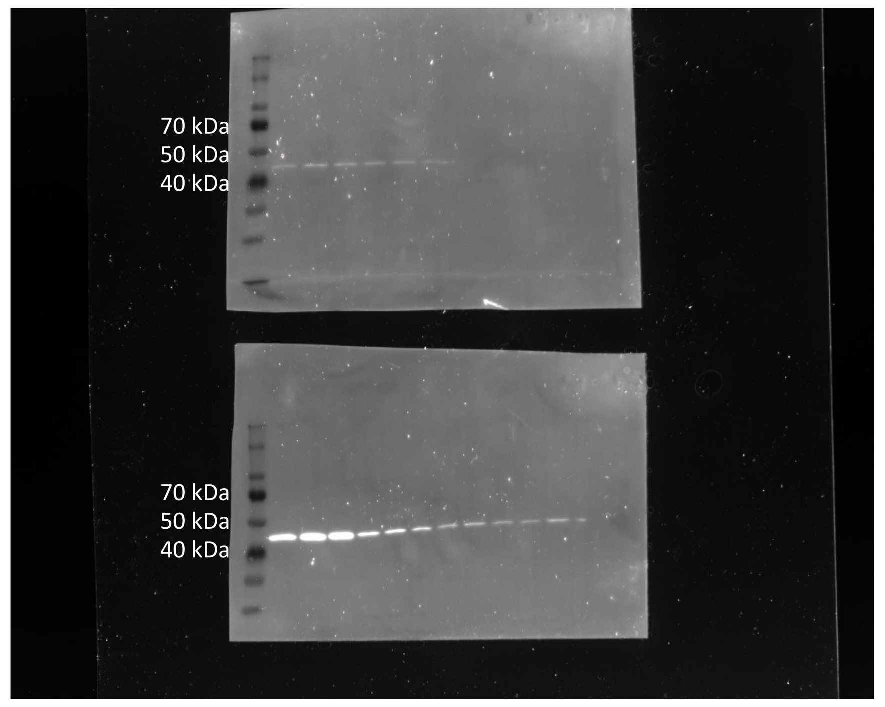

β-Tub

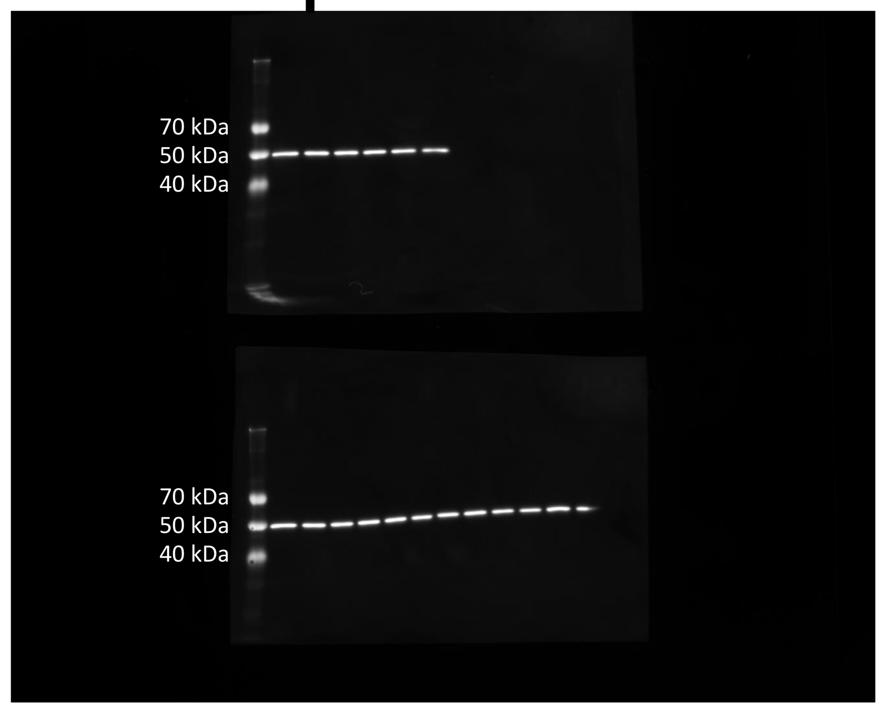

Non-Fibrotic Donor #2

αSMA

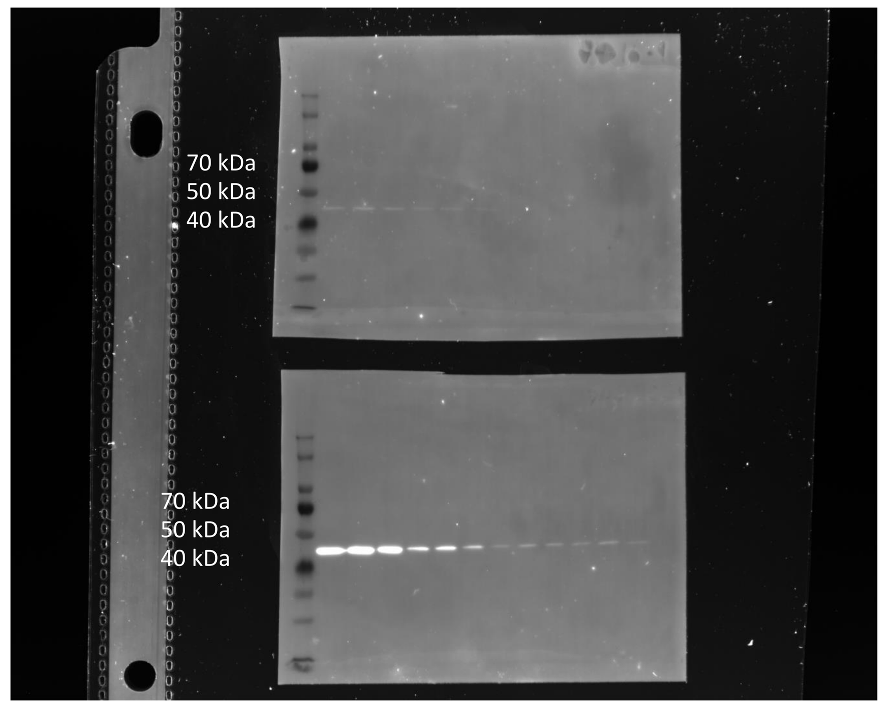

β-Tub

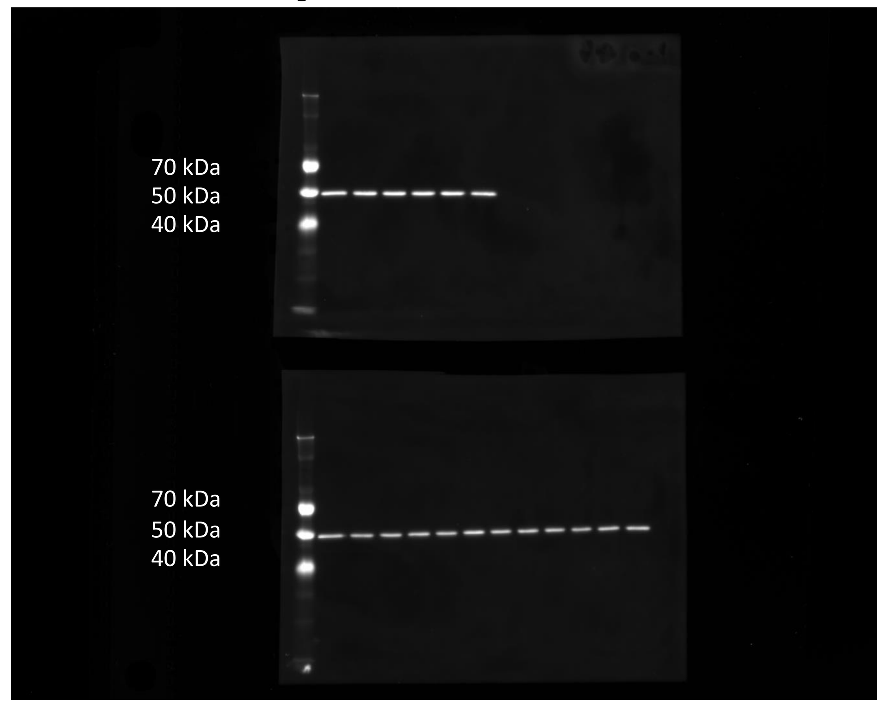

S5 Figure

Non-Fibrotic Donor #1

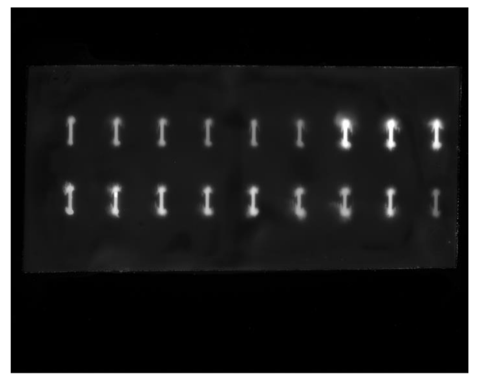

Non-Fibrotic Donor #2

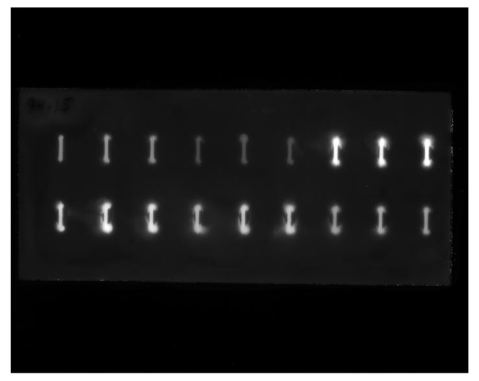

Non-Fibrotic Donor #3

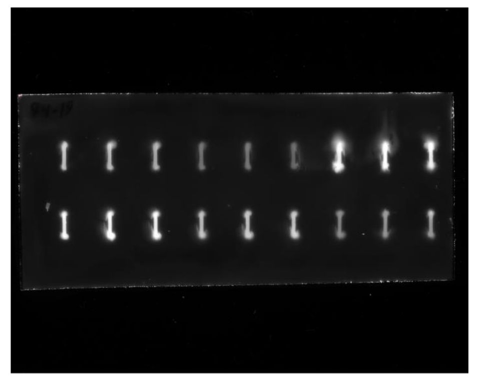

Fibrotic Donor #1

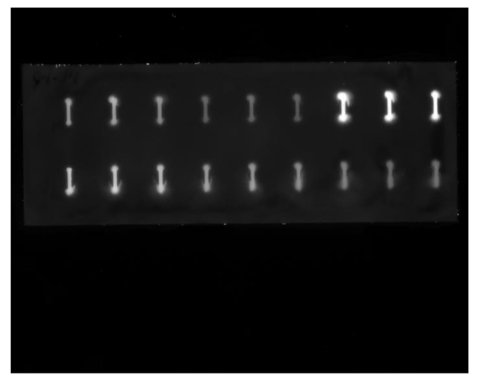

Fibrotic Donor #2

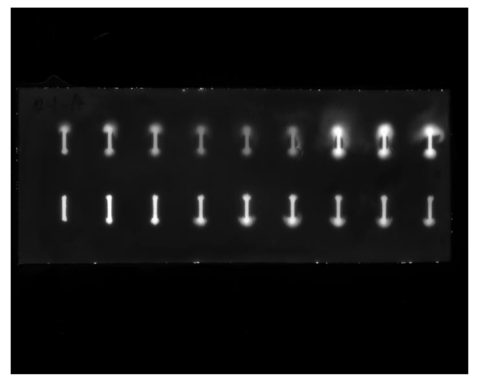

Layout for All Blots

X X X	X X X	X X X
Control	150 µM Ogerin	1 ng/mL TGF-β
X X X	X X X	X X X
TGF-β + 50 µM Ogerin	TGF-β + 100 µM Ogerin	TGF-β + 150 µM Ogerin

Figure 3A

Non-Fibrotic Donor #1

αSMA β-Tub

Fibrotic Donor #2

αSMA β-Tub

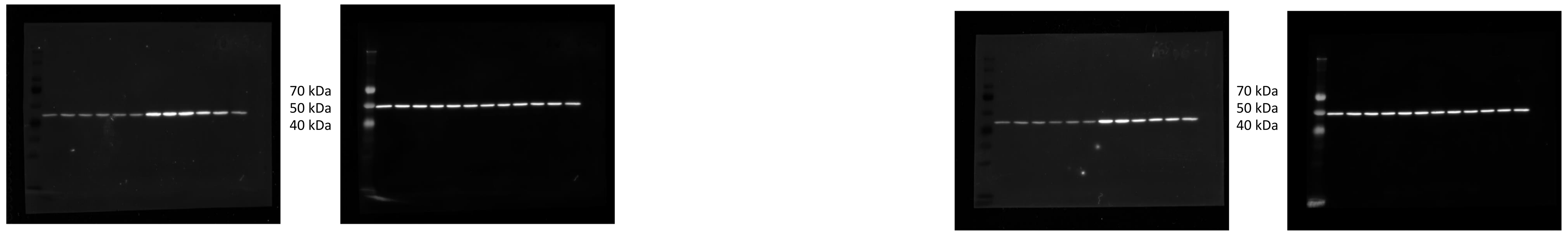

X X X	X X X	X X X	X X X
V	V	T	T
V	O	V	O

Figure 3B

Non-Fibrotic Donor #1
Fibrotic Donor #2

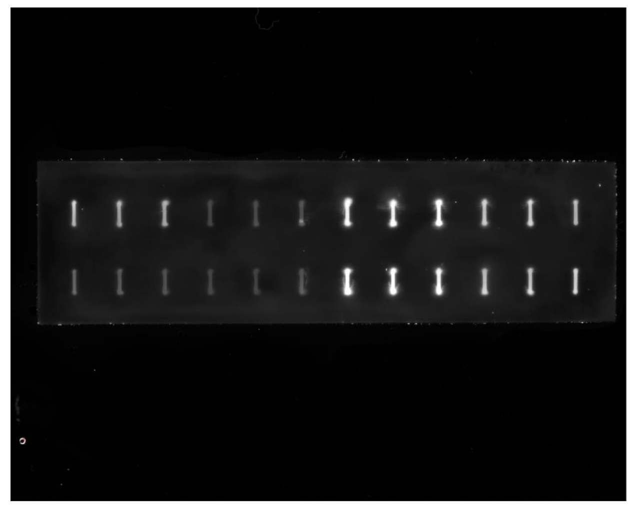

X X X	X X X	X X X	X X X
V	V	T	T
V	O	V	O

Figure 4A

Non-Fibrotic PHLFs

Cleaved PARP

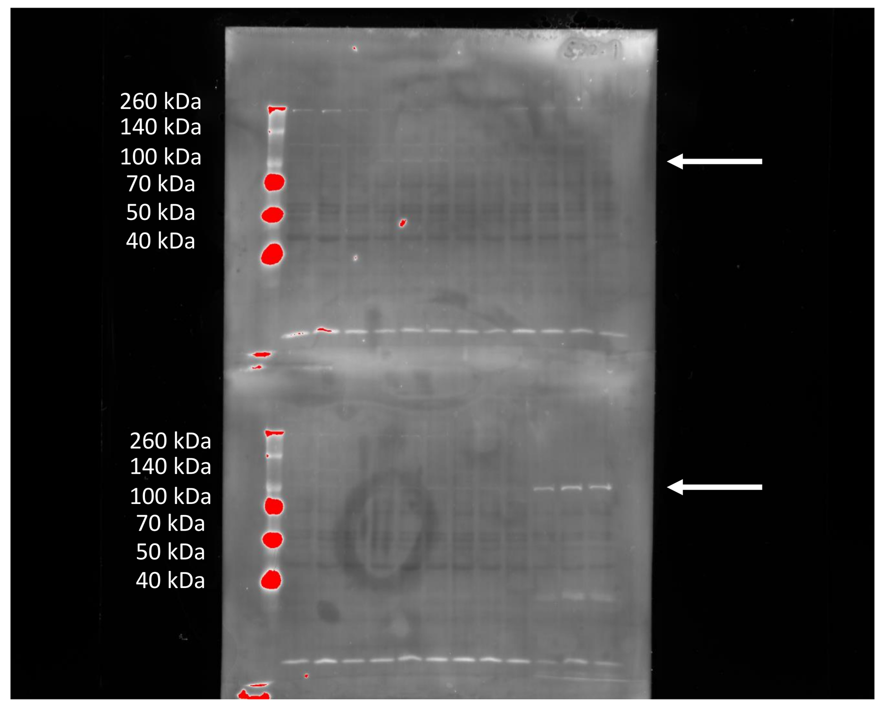

GAPDH

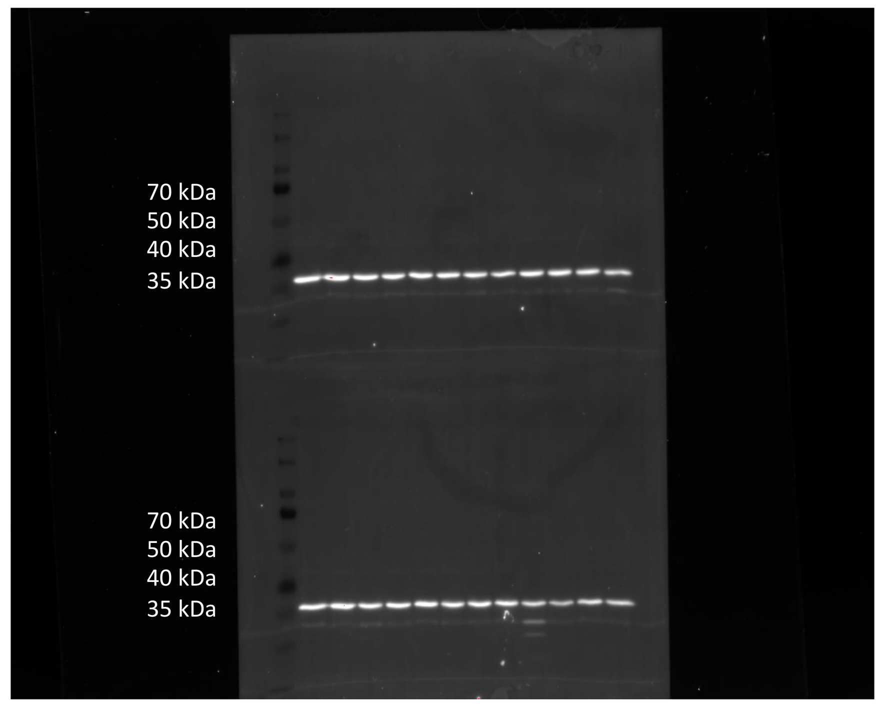

β-Tub

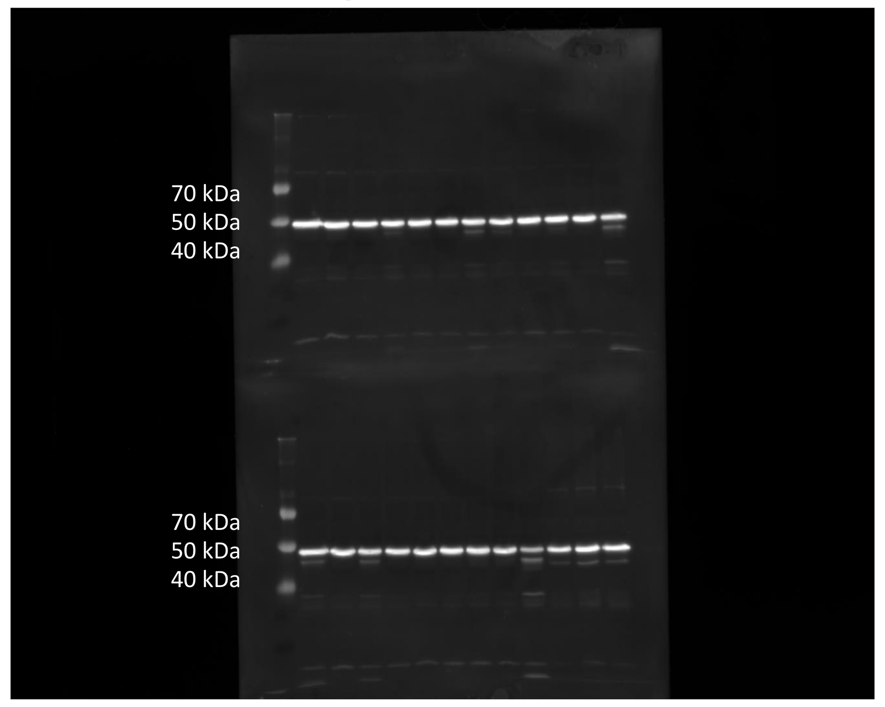

Layout for All Blots

X X X	X X X	X X X	X X X
Control	Control + DMSO	50 µM Ogerin	100 µM Ogerin

X X X	X X X	X X X	X X X
150 µM Ogerin	1 ng/mL TGFB	TGF-β + 150 µM Ogerin	5 µg/mL Puromycin

Fibrotic PHLFs

Cleaved PARP

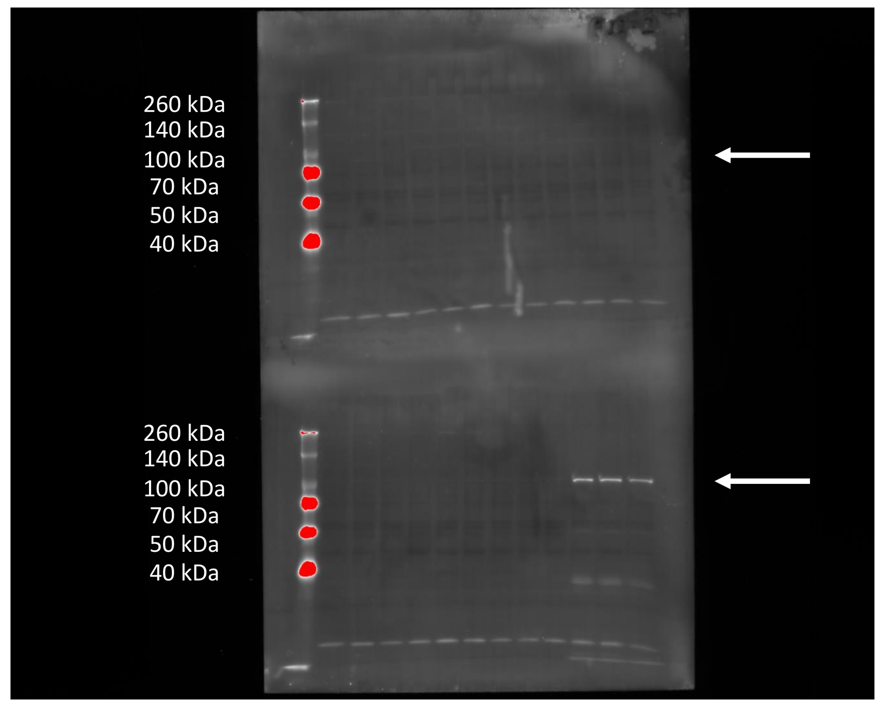

GAPDH

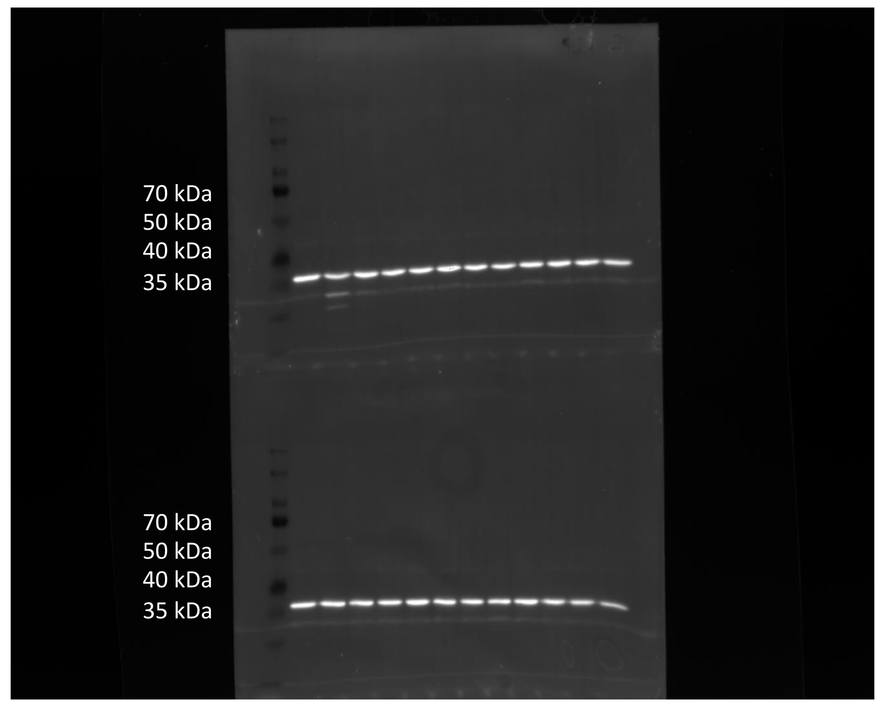

β-Tub

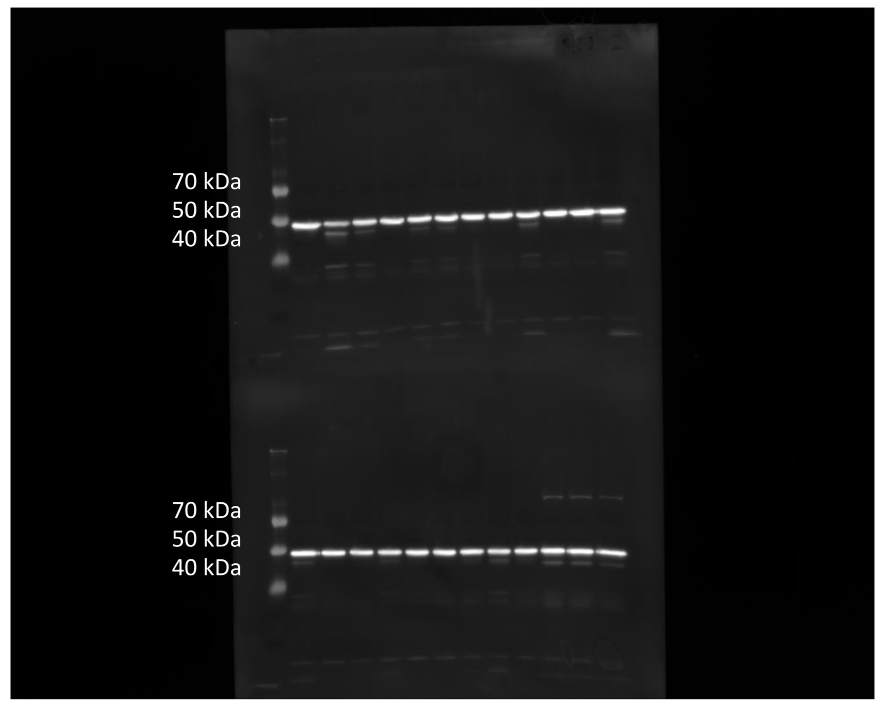

Figure 5B

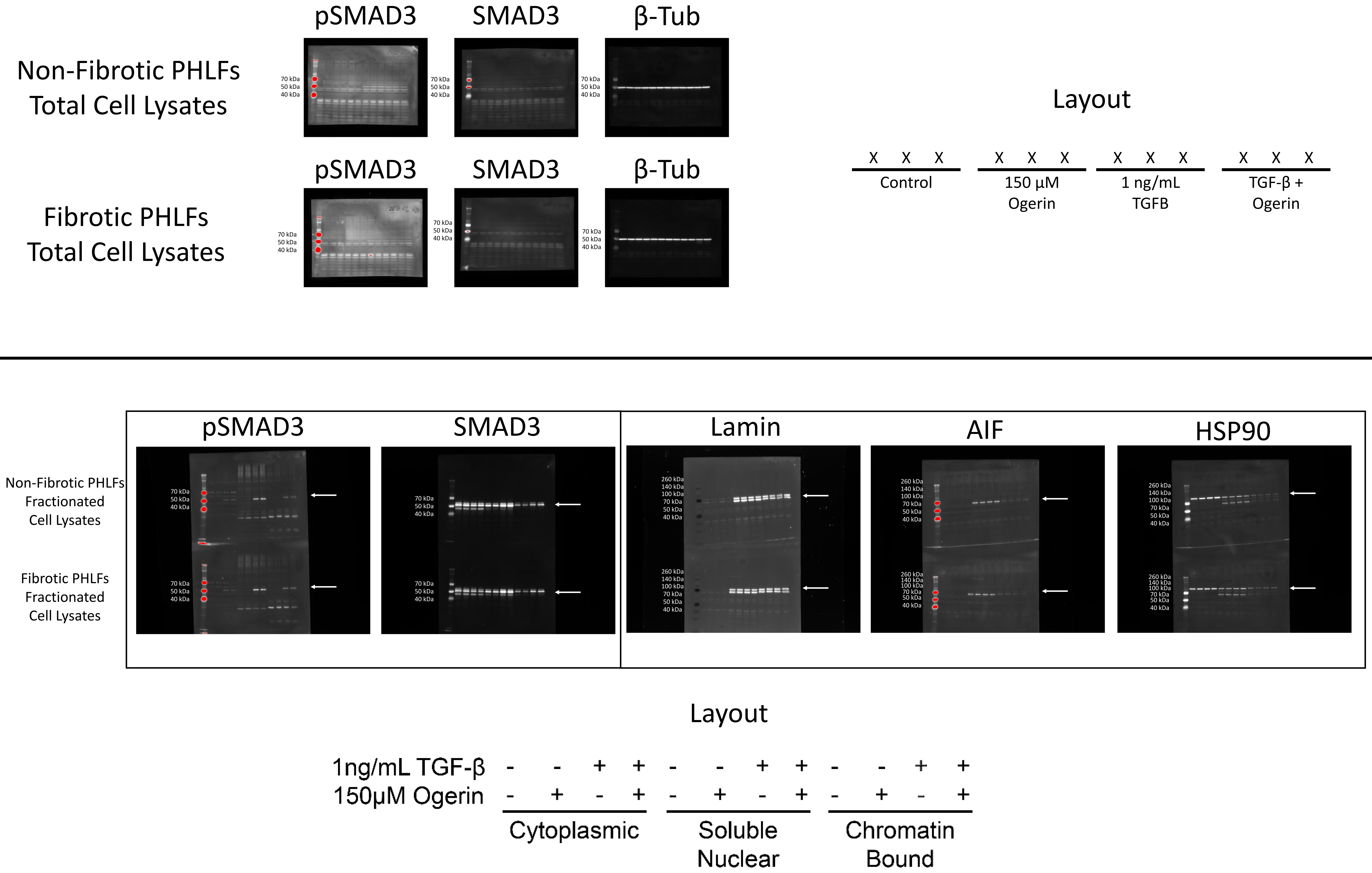

pSMAD3
SMAD3
β-Tub
Non-Fibrotic PHLFs
Total Cell Lysates
Fibrotic PHLFs
Total Cell Lysates
70 kDa
50 kDa
40 kDa
Layout
X X X
Control
X X X
150 µM
Ogerin
X X X
1 ng/mL
TGFB
X X X
TGF-β +
Ogerin
pSMAD3
SMAD3
Lamin
AIF
HSP90
Non-Fibrotic PHLFs
Fractionated
Cell Lysates
Fibrotic PHLFs
Fractionated
Cell Lysates
260 kDa
140 kDa
100 kDa
70 kDa
50 kDa
40 kDa
Layout
1ng/mL TGF-β - - + + - - + + - - + +
150µM Ogerin - + - + - + - + - + - +
Cytoplasmic
Soluble
Nuclear
Chromatin
Bound

Figure 6A

	pCREB	CREB
Non-Fibrotic PHLFs		

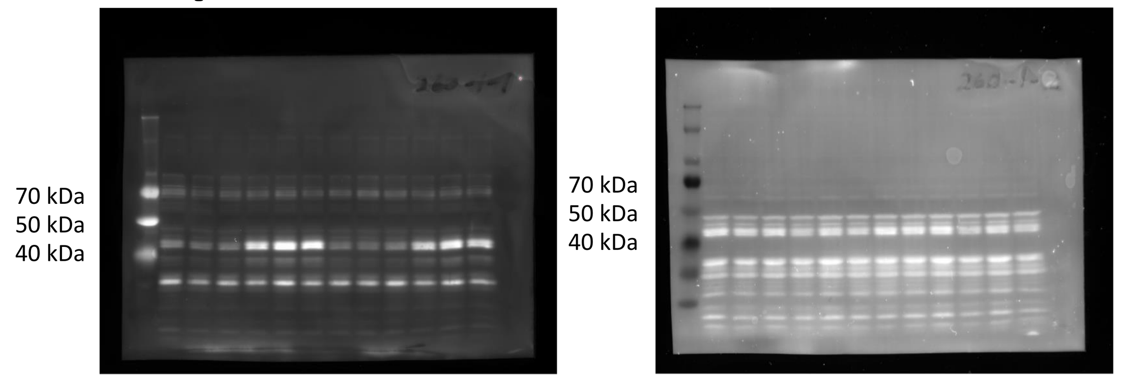

	pCREB	CREB
Fibrotic PHLFs		

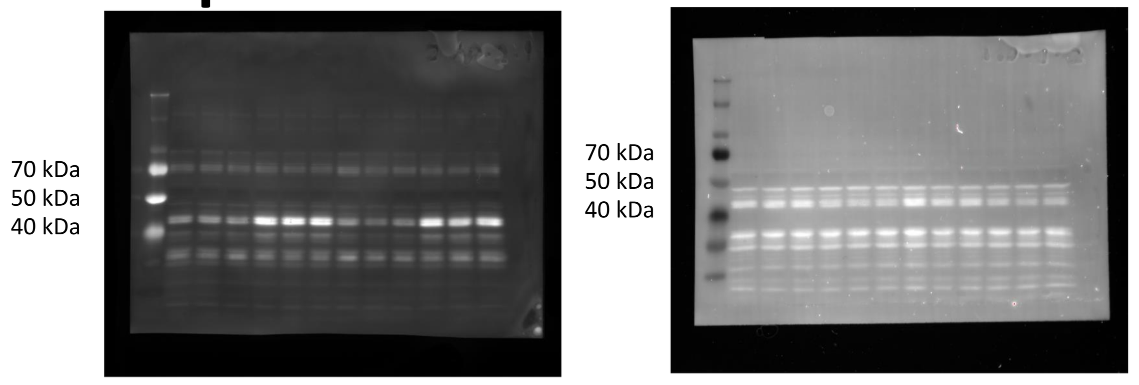

Layout

X X X	X X X	X X X	X X X
Control	150 µM Ogerin	1 ng/mL TGFB	TGF-β + Ogerin

Figure 6B

	pCREB	CREB	β-Tub
Non-Fibrotic PHLFs			

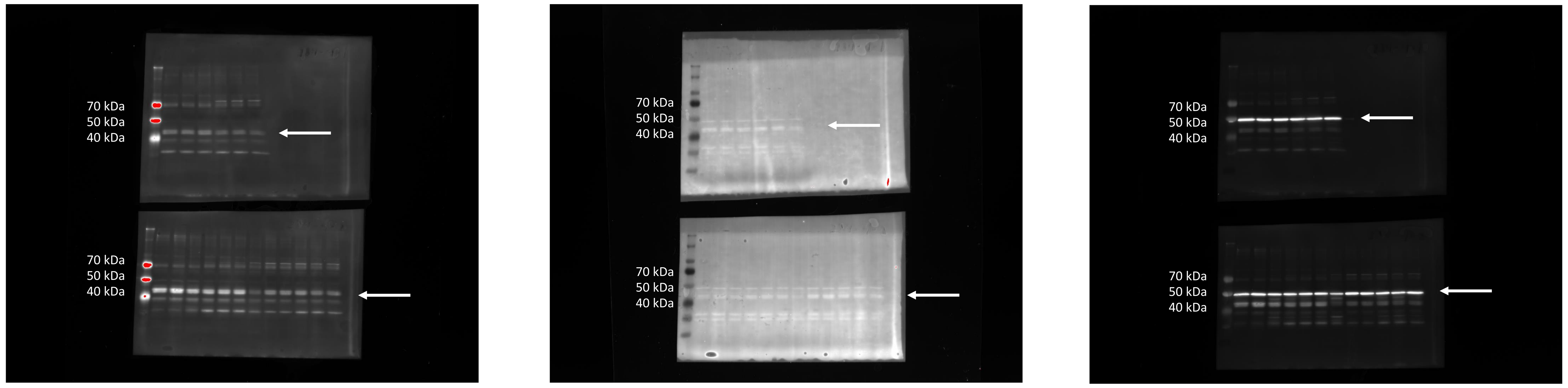

	pCREB	CREB	β-Tub
Fibrotic PHLFs			

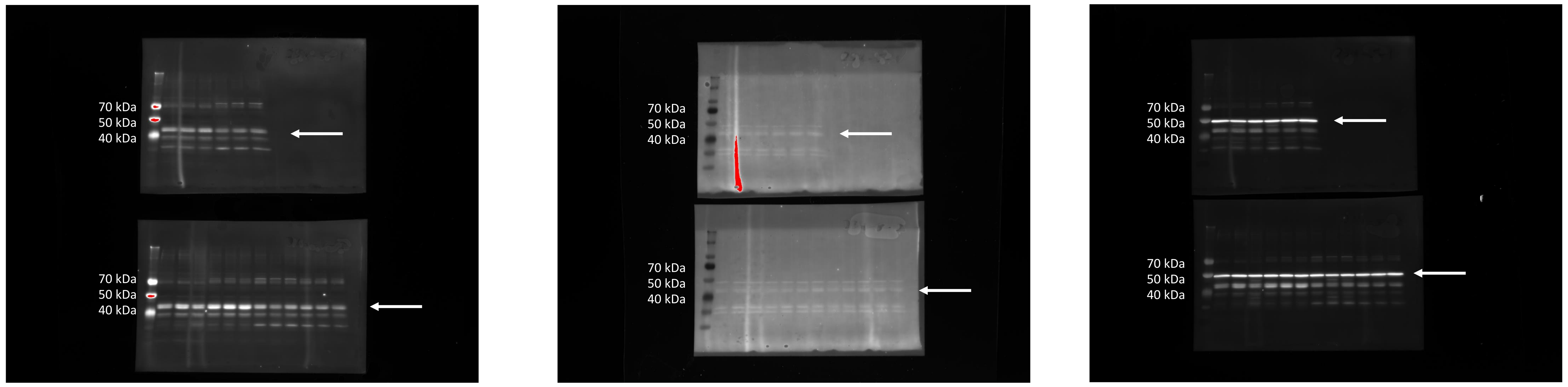

Layout

Forskolin	-	-
Ogerin	-	-
H-89	-	+

Forskolin	+	-	+	-
Ogerin	-	+	-	+
H-89	-	-	+	+

Figure 7A

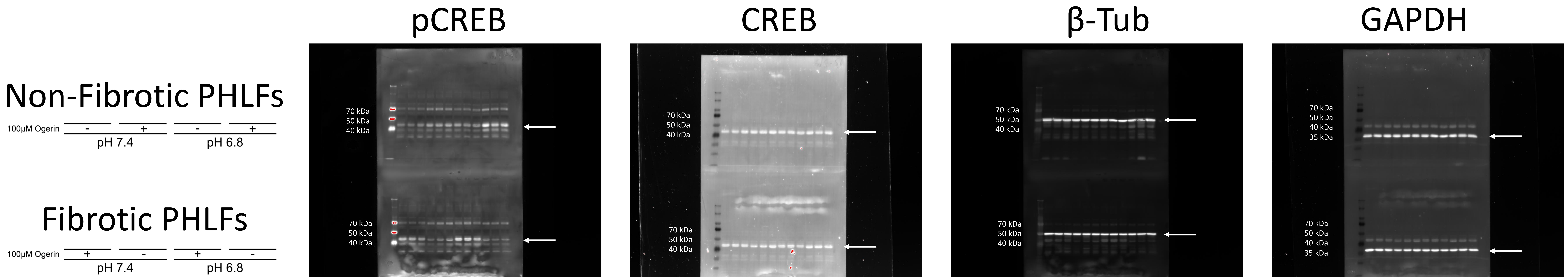

S10 Fig

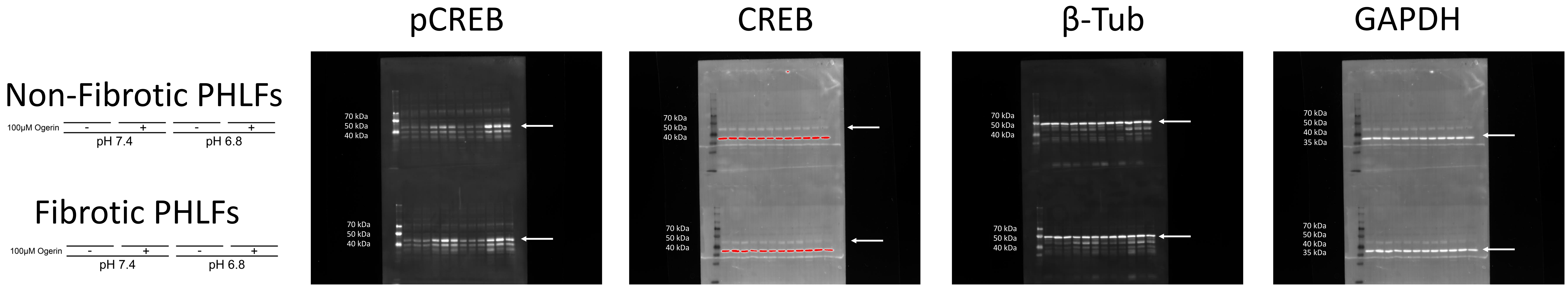

Figure 8B

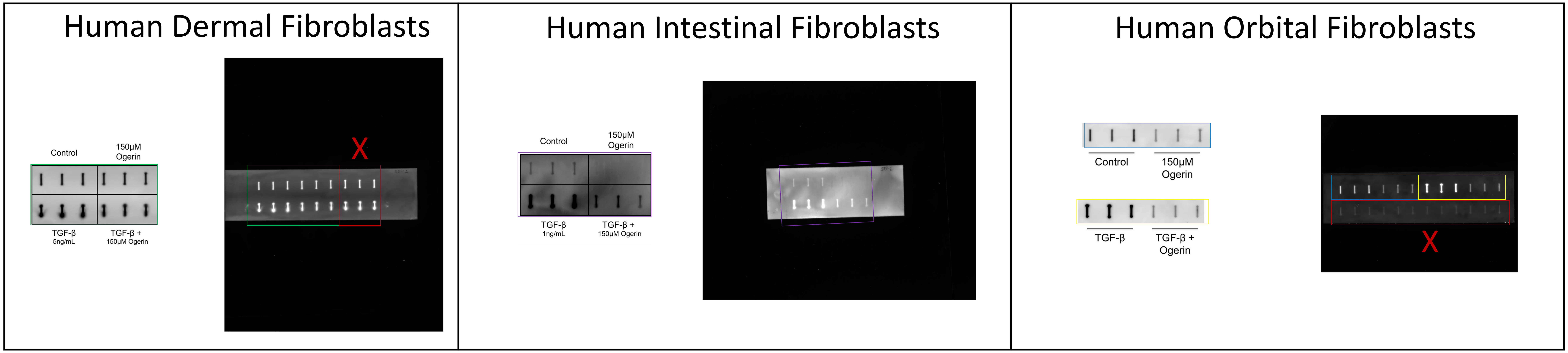

Figure 8C

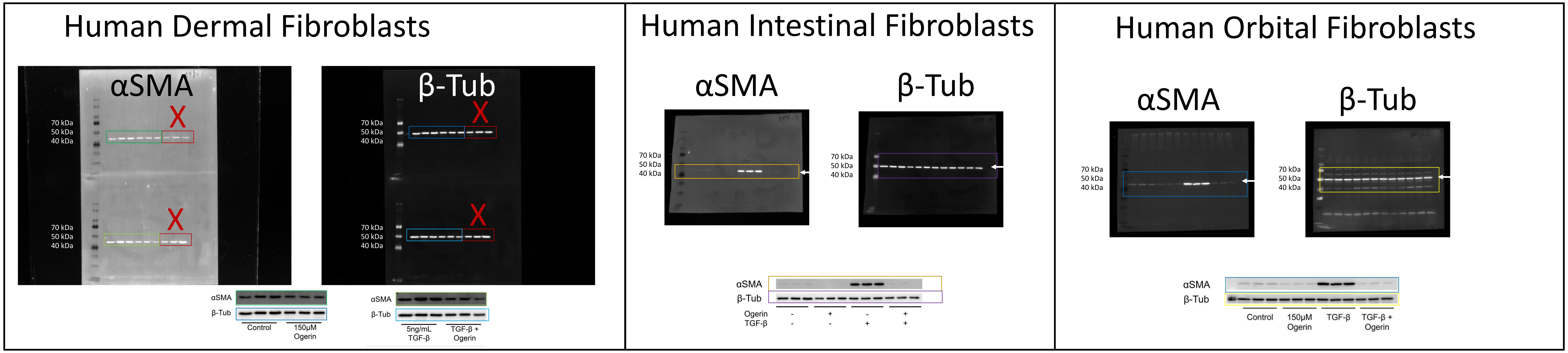

Figure 9A

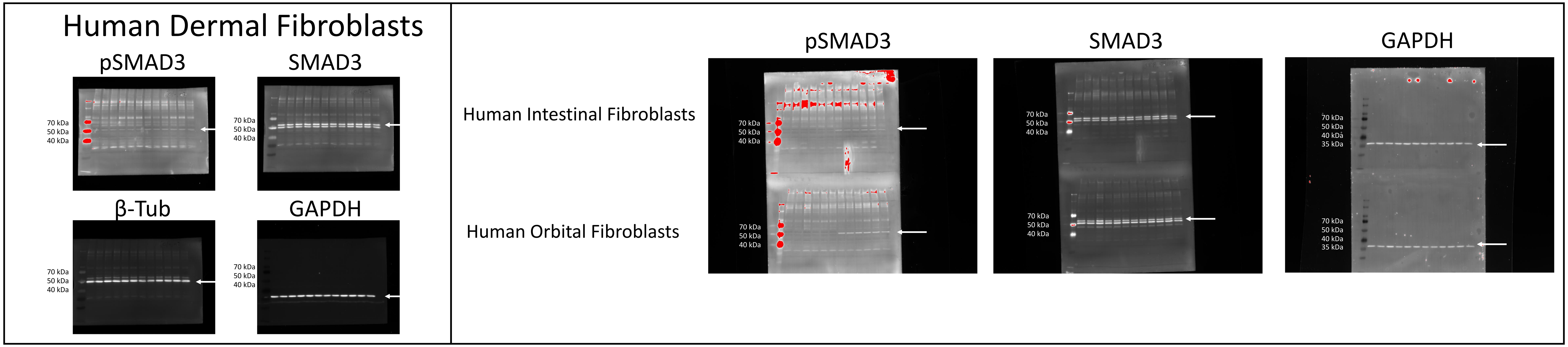

Figure 9B

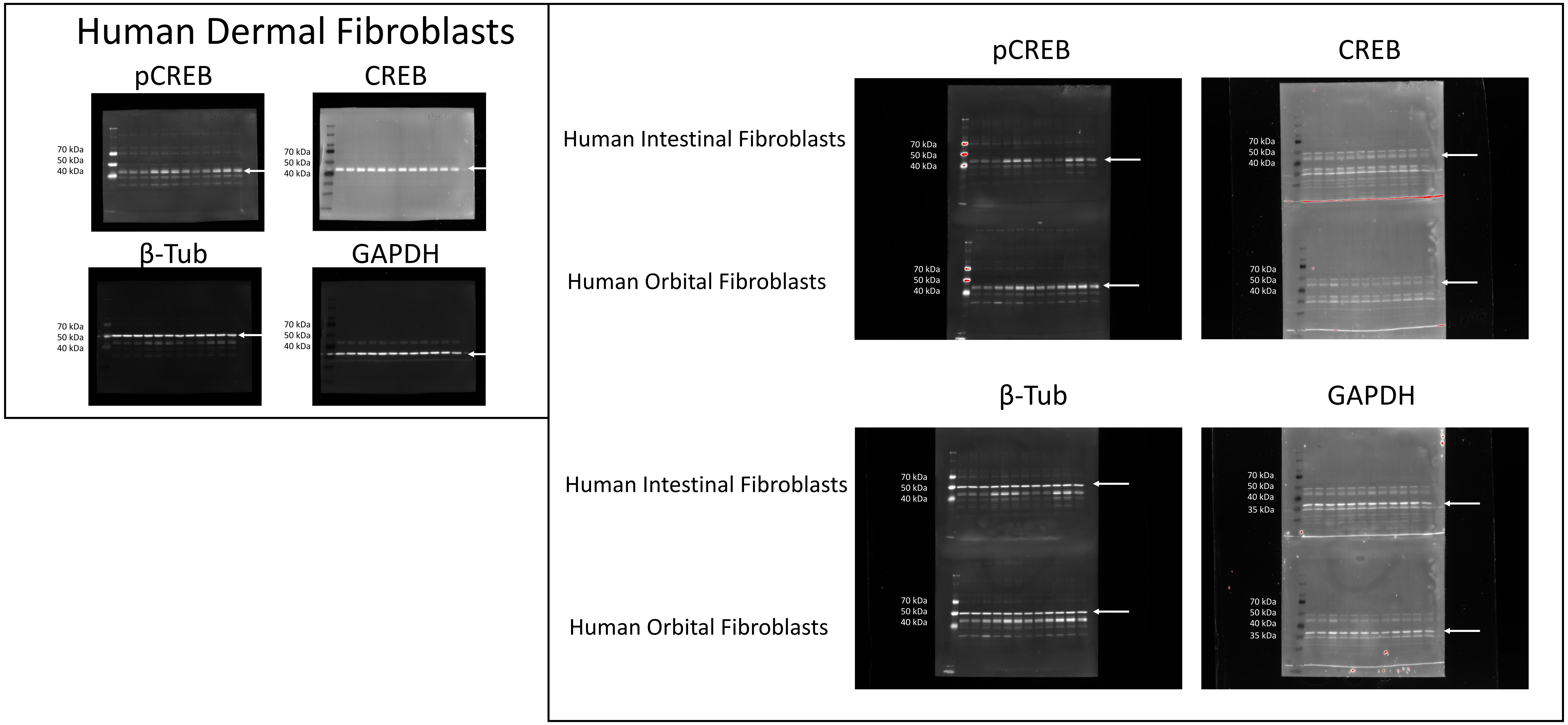

S9 Fig

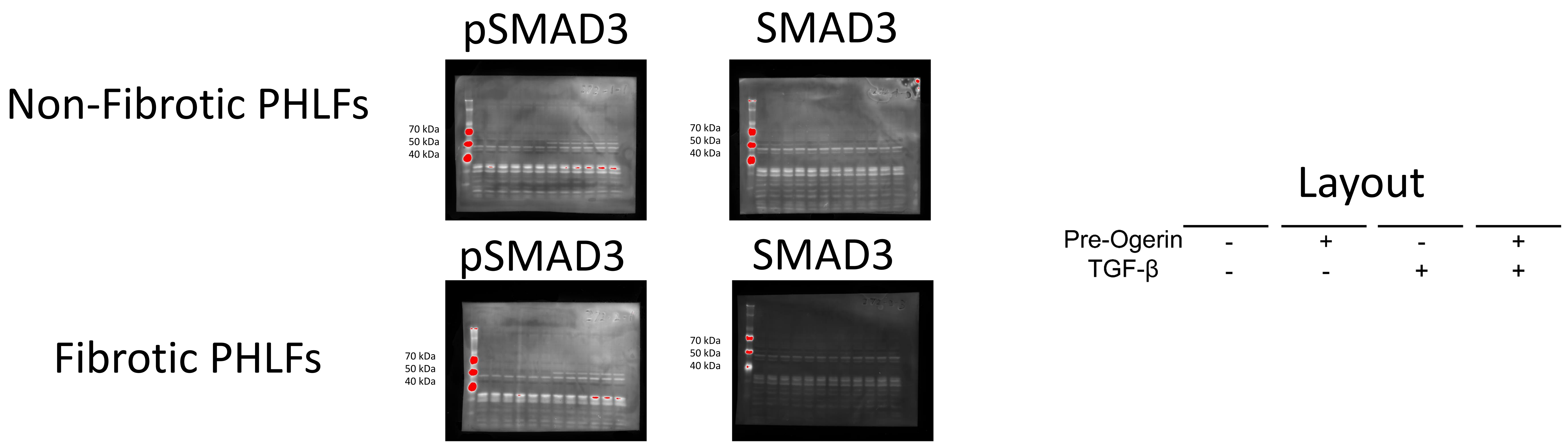
pSMAD3
SMAD3
Non-Fibrotic PHLFs
70 kDa
50 kDa
40 kDa
pSMAD3
SMAD3
Fibrotic PHLFs
70 kDa
50 kDa
40 kDa
Layout
Pre-Ogerin - + - +
TGF-β - - + +
